# Lessons from Deep Learning Structural Prediction of Multistate Multidomain Proteins—The Case Study of Coiled-Coil NOD-like Receptors

**DOI:** 10.3390/ijms26020500

**Published:** 2025-01-09

**Authors:** Teodor Asvadur Șulea, Eliza Cristina Martin, Cosmin Alexandru Bugeac, Floriana Sibel Bectaș, Anca-L Iacob, Laurențiu Spiridon, Andrei-Jose Petrescu

**Affiliations:** Department of Bioinformatics and Structural Biochemistry, Institute of Biochemistry of the Romanian Academy, Splaiul Independentei 296, 060031 Bucharest, Romania; teodofr.sulea@biochim.ro (T.A.Ș.); eliza.martin@biochim.ro (E.C.M.); alexandru.bugeac@biochim.ro (C.A.B.); floriana.bectas@biochim.ro (F.S.B.); anca.munteanu@biochim.ro (A.-L.I.); laurentiu.spiridon@biochim.ro (L.S.)

**Keywords:** protein modeling, AlphaFold, RoseTTAFold, molecular simulation, modular, multidomain proteins, coiled-coil NOD-like receptors, CNL, TNL, *Arabidopsis thaliana*

## Abstract

We test here the prediction capabilities of the new generation of deep learning predictors in the more challenging situation of multistate multidomain proteins by using as a case study a coiled-coil family of Nucleotide-binding Oligomerization Domain-like (NOD-like) receptors from *A. thaliana* and a few extra examples for reference. Results reveal a truly remarkable ability of these platforms to correctly predict the 3D structure of modules that fold in well-established topologies. A lower performance is noticed in modeling morphing regions of these proteins, such as the coiled coils. Predictors also display a good sensitivity to local sequence drifts upon the modeling solution of the overall modular configuration. In multivalued 1D to 3D mappings, the platforms display a marked tendency to model proteins in the most compact configuration and must be retrained by information filtering to drive modeling toward the sparser ones. Bias toward order and compactness is seen at the secondary structure level as well. All in all, using AI predictors for modeling multidomain multistate proteins when global templates are at hand is fruitful, but the above challenges have to be taken into account. In the absence of global templates, a piecewise modeling approach with experimentally constrained reconstruction of the global architecture might give more realistic results.

## 1. Introduction

There is a general consensus that structural biology has entered a new era with the advent of the new generation of deep learning predictors such as AlphaFold and RoseTTAFold [1,2,3,4,5,6]. However, how these predictions relate to the complex reality that proteins face in their cellular environment is still an open question. Proteins are complex molecular objects displaying tightly controlled structural progression into the cell during their turnover—from nascent to native to denatured, finally degraded states. Only ~5% of them are able to refold in solution, while the rest need interactions with chaperones to guide their folding into the native state, meaning that their structure is intrinsically constrained and depends on the environment [7,8]. Moreover, between 60% and 80% of a proteome comprise sequences with multiple structurally and functionally distinct modules, including intrinsically disordered regions [9,10], with their complexity increasing along the tree of life [11], as such modular connectivity ensures optimized hierarchical molecular function bundling is driven by a significant increase in local encounter rate [12,13]. Delineating domains is not always trivial, as only a few multidomain proteins fold into a beads-on-a-string discrete manner while the rest display more or less extensive interdomain interfaces and intricate, hard-to-distinct connecting regions generating an almost continuous domain overlap into the folding space [14], which causes significant delineation differences between the main domain classification methods: CATH [15], SCOP [16], SCOPe [17] and ECOD [18]. Further complications arise from the fact that the functional structure of many multidomain proteins depends on the integration or exchange of cofactors [19] or on external interactors inflicting stern structural transitions during their functional cycle, for instance, in processes such as signal transduction or viral cell entry [20,21]. Thus, it is legitimate to ask what the limits of the new deep learning technologies are in protein structural prediction.

A good example for assessing AI prediction capabilities on more complex multidomain proteins is the family of coiled-coil NOD-like (CNL) receptors on which, over the past decade, we gained significant insight using a computationally aided experimental approach [22,23,24,25,26,27,28,29,30,31,32]. CNL receptors are involved in the so-called effector-triggered immunity (ETI) and display many of the above-mentioned complexities. The ‘core’ of a CNL sequence contains three main modules: a ‘signaling/connector’ CC domain, an NBD ‘switch’ and an LRR ‘sensor’. Of these, NBD further consists of two blocks: NBS-Arc1 and Arc2, which suffer a 180° rotation with respect to NBS-Arc1 upon CNL activation, induced by its interaction with a pathogen effector protein. This further generates an overall change of the receptor structure accompanied by a cofactor exchange from ADP in the inactive state to ATP in the active state. In some CNL extensions, this ‘core’ region may arise toward both the N- and C-terminal regions referred to as ‘integrated domains’ [33]. Recently, the structure of one member of this family from *Arabidopsis thaliana*, HOPZ-ACTIVATED RESISTANCE1 (ZAR1), has been solved in its inactive, intermediate and active final state in which a pentamer resistosome is formed that protrudes the cell membrane and leads to cell death [34,35]. However, even if all CNLs maintain the same canonical domain organization, other members of this family were shown to perform other functions [36], such as those related to the defense gene activation via interactions with transcription factors [37] or to the immune signaling by various types of sensor and helper CNLs that (re)act as singletons, pairs or networks [38].

Herein, we use the best-known AI prediction methods: AlphaFold2 (AF2) [39], AlphaFold3 (AF3) [40] and RoseTTAFold All-Atom (RFAA) [41] to build 3D models of 32 representatives selected from the CNL family from *Arabidopsis thaliana* and analyze them by both assessing their standalone quality and with up-to-date available experimental data.

## 2. Results

### 2.1. Gauging the Modeling Performance of AI Platforms by Using ZAR1-Solved Structures

As the structure of the *A. thaliana* ZAR1-CNL receptor was recently solved by Cryogenic electron microscopy (Cryo-EM) in its inactive, transition and active states, assessment of the predictive capacity of AF2, AF3 and RFAA was performed directly by comparing models obtained in various training conditions (described under Section 4) with the experimental data both globally and on a domain-by-domain basis.

#### 2.1.1. Model Performance at Local Domain Level

At the domain level, the C_α_ RMSD of all models against active and inactive experimental structures are shown in Table 1 and Table 2, respectively. It is interesting to note that while predictions of NBD and LRR were, in all cases, of very high quality, most models presented severe departures from the experimental data in the CC domain region, with RMSDs of over 12 Å. Only by using a trivial “AF2—Active/Inactive control” workflow can a better representation of all ZAR1 modules be obtained. However, this is not significant since this workflow only uses ZAR1 active/inactive templates to train the local implementation of AF2 and eliminate the multiple sequence alignment (MSA) step that AF2 performs by default. By allowing the MSA step as well, the prediction quality of the CC severely degrades to the same level as the rest of the models.

**Figure 1:**
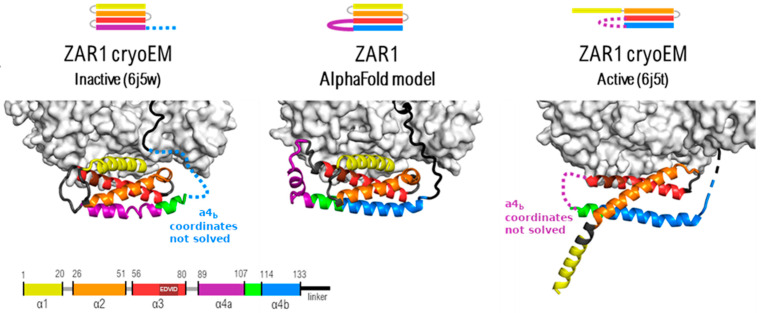
ADP, ATP and no ligand, respectively. Crystal structures of active (**right**) and inactive (**left**) versus the AlphaFold Database model (**center**). A simplified representation of each state is presented, showcasing how the AF2 model combines the two existing structures.

At closer inspection, the local modeling solution proposed by all platforms for the CC region is basically a four alpha-helix bundle, which mixes up segments modeled according to the active and inactive state templates, respectively, as shown in Figure 1.

**Figure 2:**
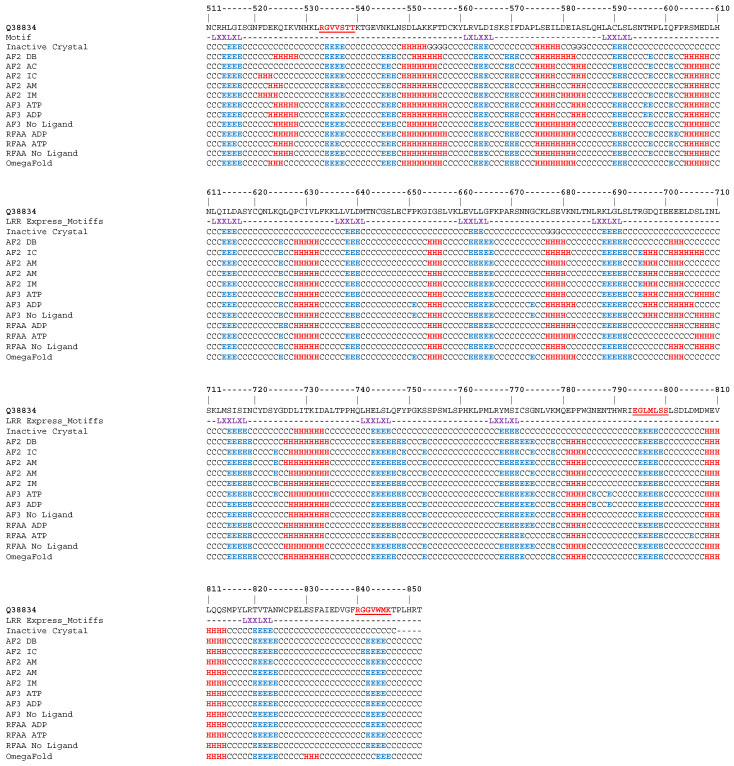
Alignment of secondary structure profiles of all models generated for ZAR1 and of the inactive crystal. Model names have been abbreviated as follows: AF2 DB: AlphaFold2 Database; AF2 AC: AlphaFold2 Active Control; AF2 IC: AlphaFold2 Inactive Control; AF2 AM: AlphaFold2 Active MSA; AF2 IM: AlphaFold2 Inactive MSA; AF3 ATP/ADP/No Ligand: AlphaFold3 model with ATP, ADP and no ligand, respectively; RFAA ADP/ATP/No Ligand: RoseTTAFold all-atom model, with ADP, ATP and no ligand, respectively; OmegaFold: OmegaFold. The underline sequence residues highlight areas which adopt a beta strand structure, but do not feature typical LxxLxL motifs (which are marked in purple).

Mapping the secondary structure over the LRR domain indicates that all models accurately reproduce most of the structural features. As can be seen from Figure 2, the extended beta strands forming the beta-sheet ventral structure of the LRR domain consistently match that derived from Cryo-EM data. In contrast, on the dorsal side of the horseshoe, all models display a highly biased helical tendency when compared to the experimental data. Coming back to the ventral region, it is interesting to note that not all beta strands forming the beta-sheet network were typical LxxLxL motifs as those found in the regions 533-RGVVSTT-538, 794-EGLMLSS-800 and 840-RGGVWMK-846.

#### 2.1.2. Prediction Performance at the Global Architectural Level

The global RMSD of the highest-ranked models are presented in Table 3. As expected, the lowest RMSDs were shown by the trivial “AF2—Active/Inactive control” models due to the severe restrictions imposed by the input filtration and curation of structural information. As can be seen closer to the experiment was the so-called “AF2—Active MSA” model, which, besides relying only on the active specific templates, also included the AF2 MSA step. This is significant as it indicates that feeding AI modeling with specific structural 3D information might improve, in some cases, the prediction quality of the global architecture of multistate multidomain proteins, at least in high sequence homology cases.

**Figure 3:**
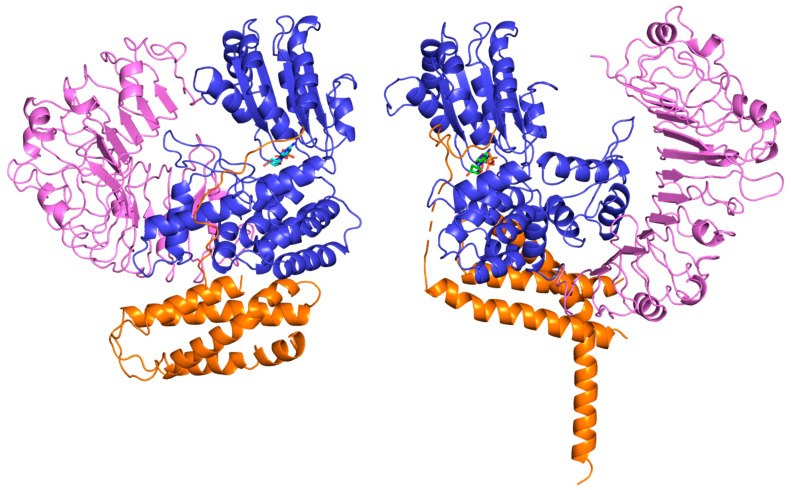
Comparison between the solved structures of the sparser active (ATP-bound) state (**left**) and the more compact inactive (ADP-bound) state (**right**). For each structure: CC domain—orange, NBS domain—blue, LRR domain: pink.

As can also be seen from Table 3, when based on the default training input (i.e., based on the complete structural information including all NBD templates in any of their active, inactive or transition states), all AF2-DB, all-AF3s and all-RFAAs models were closer to the inactive ADP compact domain configuration (<6 Å) rather than to the active ATP model (>20 Å) in which the domains were sparse (Figure 3).

Moreover, the ~6 Å RMSD is largely due to the CC modeling problems, as discussed above, rather than to departures from the overall configuration. It is highly remarkable that even when modeling ZAR1 in the presence of ATP—which is specific to the ‘open’ active state of both AF3 and RFAA—it models the protein in its inactive ADP compact configuration. These models feature the ATP correctly located in its binding site but placed in the wrong overall architecture, which means that ligand information is not taken into account in modeling the protein moiety.

Model stability was tested by molecular dynamics (MD) simulations. To this end, all models were first subjected to energy optimization, and those showing the lowest final potential energy were selected for classical MD runs, as described in Section 4. None of the selected models unfolded during this test or displayed any significant local structural departures, while the RMSD remained low, similar to that of the experimental models, as can be seen from Supplementary Table S3.

**Figure 4:**
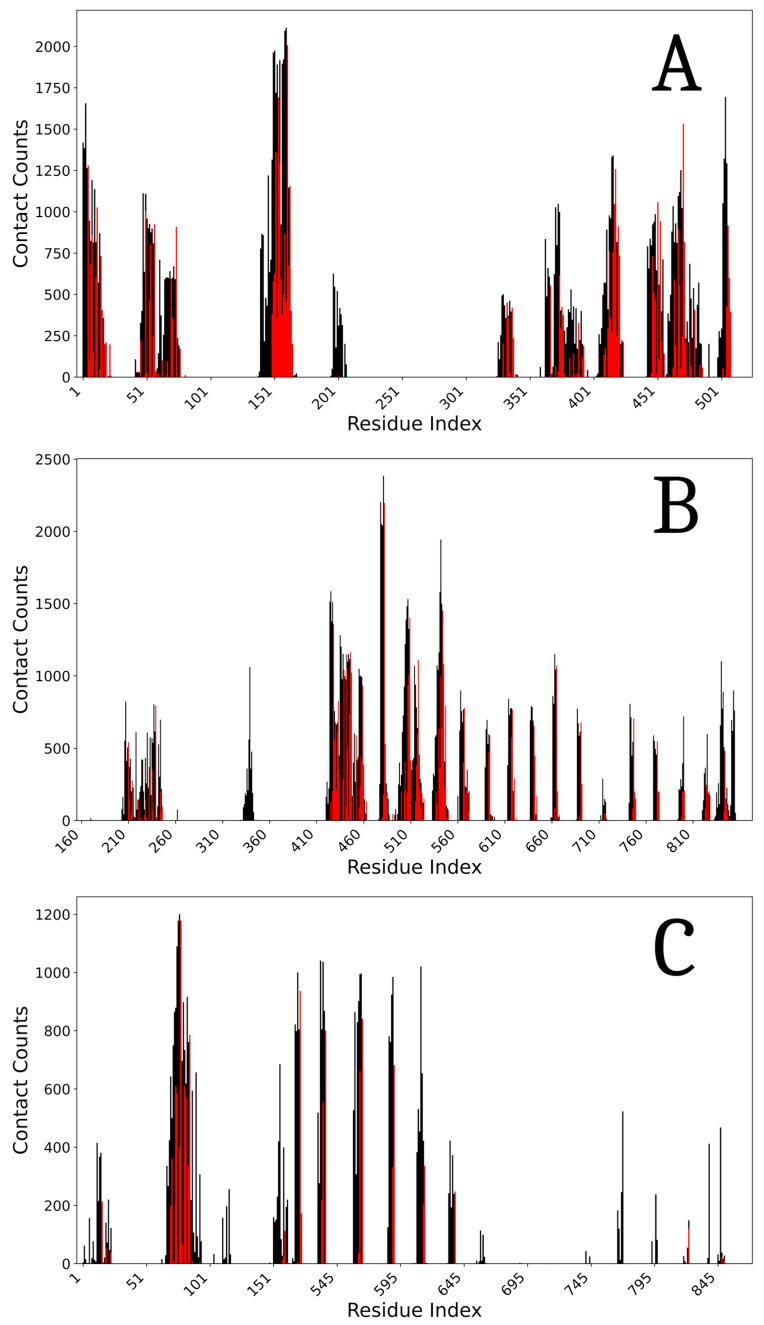
Bar plots for each inter-domain pair for the inactive conformations. The black bars show how many times a residue in each domain interacts with a residue in the corresponding domain, for the generated models. The red bars are contacts featured in the experimentally solved structure. (**A**): CC-NBS; (**B**): NBS-LRR; (**C**): CC-LRR.

Gauging the modeling performance in interdomain interface predictions is also significant. As discussed, except for the AF2 filtered workflows designed for targeted active/inactive modeling, the default AI platforms modeled ZAR1 in the ‘closed’, inactive ADP state, even when modeled in the presence of the active state ATP ligand. Detailed analysis indicates that all models reproduced the experimental interdomain interfaces of this compact state well. Interestingly, even if the local modeling solution proposed for the CC highly diverges from the experiment, all platforms correctly predict its interface with the LRR domain. The agreement between models and the inactive ADP experimental structure is indicated by MD-based free energy estimations and, in more detail, by the interdomain contact histograms shown in Table 4 and Figure 4, respectively. The slightly tighter (~5 ÷ 10%) binding shown by models vs. experiment results from an average 16% increase in the number of contacts, which also indicates a model tendency toward compacting the structures.

### 2.2. Modeling the CNL Set

#### 2.2.1. Sequence Selection

The original set of 1257 sequences retrieved from NLRscape [42] was subjected to a filtering procedure described in Section 4. In the first round, sequences lacking NBD and those with TIR signatures at the N-terminal end were eliminated. This reduced the set to 376 predicted CNLs. In the second step, any sequence lacking any of the nine canonical NBD motifs was eliminated using NLRexpress [43], resulting in a 256 set of predicted fully functional CNL sequences. This was further subjected to clustering at 70% sequence identity and 70% sequence coverage using MMSeqs2 v15.0 [44], resulting in 36 groups, given in Table 5. From these, the most cited and experimentally used sequence was selected as representative of each cluster and was used further. However, four clusters were eliminated from the analysis, the first (#16) due to the fact that its members have no citations and present extra marginal domains, the second (#21) because the cited member had extra domains, the third (#25) due to the fact that its sequences were unusually short (<675 aa) and the fourth, sequence A0A5S9WPD4, was due to missing parts of the CC and LRR domain of A0A1P8AP86, which has a > 75% identity on the rest of the common frame.

With these, the set of 32 *A. thaliana* CNL representatives shown in Table 5 were retained for further analysis.

**Figure 5:**
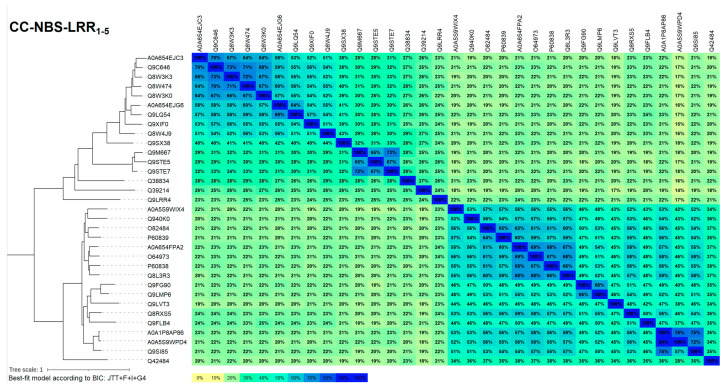
Similarity tree and matrix for the 32 sequences modeled further, as well as for the A0A5S9WPD4 sequence, which was eliminated (due to high similarity to A0A1P8AP86).

The similarity tree and the similarity matrix of this set are shown in Figure 5, while the annotated global alignment is available in Supplementary Figure S1. As can be seen, the tree has two main and even branches corresponding to two main types of CC do-main—a group of 16 EDVID motif CC type CNLs and a group of 16 RPS5-like CC type helper CNLs. Interestingly, the first ‘EDVID’ branch is sparser, with similarity levels var-ying from 20 to 40–50%, while the ‘RPS5’ branch is far more compact, with all similarity levels higher than 40%.

#### 2.2.2. Model Generation

The models retrieved from the AF2 Database.v4 (latest release at the time of writing), those from the AlphaFold3 Server and from the NeuroSnap [45] implementation of RoseTTAFold All-Atom, along with all models generated by the local deployment of AlphaFold2 are available for download at https://github.com/Teohoho/CNL_Paper_Data (created on 26 November 2024).

#### 2.2.3. Model Refinement and Analysis

**Figure 6:**
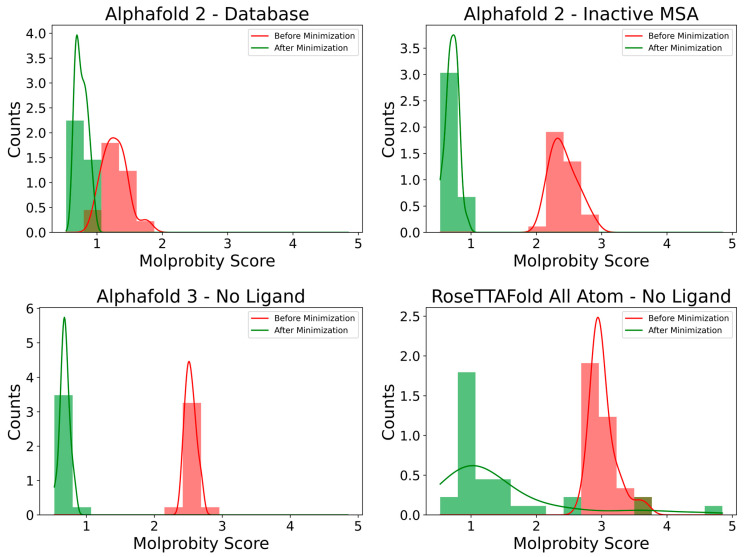
Histogram of pre- and post-minimization distributions of MolProbity scores for a subset of the generated models.

All models were minimized using the default L-BFGS algorithm as implemented in OpenMM [46]. Pre-optimization and post-optimization model quality was assessed with MolProbity [47], and the results are shown in Supplementary Tables S1 and S2. The overall distribution of pre- and post-minimization Clash Score for a subset of the models is shown in Figure 6. As can be seen, global energy minimization results in a significant quality improvement in the case of AF2 and AF3 models, while the NeuroSnap-generated RFAAs still lag behind with a large Clash Score—indicating potential entanglements and significant compactness. As RFAA models display this trend both in the presence and absence of ligands, it suggests that these small molecules are not the root of the low score for the NeuroSnap RFAA model problem.

In trying to rescue these models, simulated annealing was used to further optimize them. Only some of these models were able to withstand this step while attempting to heat the rest, leading to severe numerical instability, making them unfixable via the described annealing protocol. For this reason, the RFAA models were not simulated further.

For the highest-ranked AF2 and AF3 models, stability was evaluated using GCHMC simulations performed with Robosample [48], as described in Section 4. The highest RMSD (Å) obtained during the simulation is presented in Supplementary Table S4. Results indicate that all CNL models are stable, do not unfold globally or locally and are confined in a configurational basin not larger than 2 Å RMSD, on average.

#### 2.2.4. Conformation Preference

**Figure 7:**
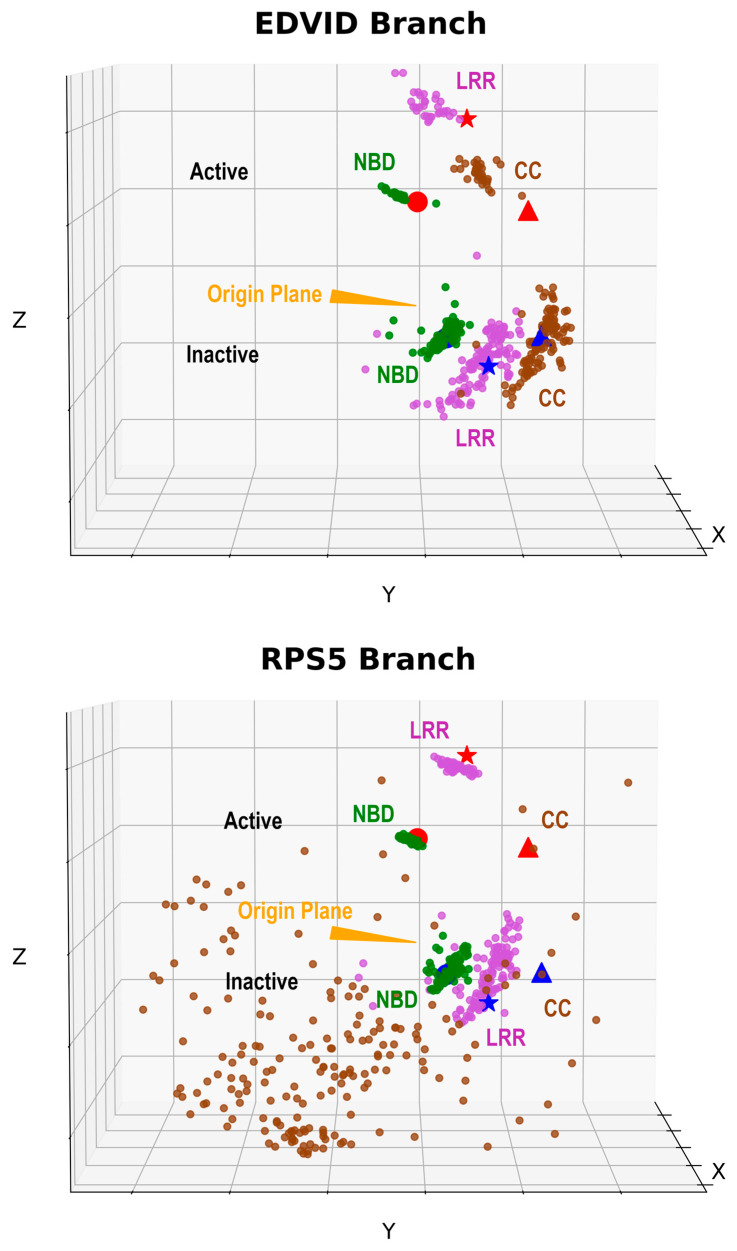
Distribution of CC (brown circles), Arc2 (blue circles) and LRR (purple circles) domains of the modeled sequences, and the same domains of the experimental structures (red circle: active Arc2 center, red triangle: active CC center, red star: active LRR center; blue circle: inactive Arc2 center, blue triangle: inactive CC center, blue star: inactive LRR center). The orange triangle represents the “VGG—NBS center—Arc1 center” origin plane, as described.

As described under Section 4, a simplified representation of the CNL configuration was set for each model in a reference system centered in the alpha carbon of the glycine of the “VVG” NBD entry motif, with the xOy plane generated with this origin and the NBS and Arc1 domain mass centers. This plane consistently separates the ADP-inactive and ATP-active NBD configurations. Figure 7 displays the distribution of CC, Arc2 and LRR1-5 mass centers for a subset of models. The full domain assignment is given in Supplementary Table S3. As can be seen, all models on the ‘EDVID’ CNL branch are generated in either ADP-inactive or ATP-active configurations with a bias toward the compact ADP-inactive structure. It also shows that modeling CNLs in the presence of ATP does not affect the preference toward the closed, compact ADP form, indicating that information regarding ligand specificity is not taken into account by either AF3 or RFAA AI platforms in modeling the protein moiety. Interestingly, in the absence of ADP or ATP, the AF3 predictor models the sequences closer to the sparse, active CNL configuration. Turning now to the second ‘RPS5-like’ CNL branch, all models display a sparse CC configuration, completely different from that shown by the ‘EDVID’ group and documented by the ZAR1 Cryo-EM data—represented by the cluster of CC domains in the bottom left of each 3D scatter plot. A more detailed analysis reveals that on this branch, the CC does not interface anymore with the rest of the CNL, suggesting a completely different mechanism of signaling or multimerization.

In order to see if results hold for CNL families from other species, several well-studied receptors from wheat, barley and potato were also subjected to the same modeling protocols. Over 75% of these further examples were modeled in the ADP inactive, compact state, as can be seen from Supplemental Table S5, which presents the domain coordinates corresponding to Figure 7.

**Figure 8:**
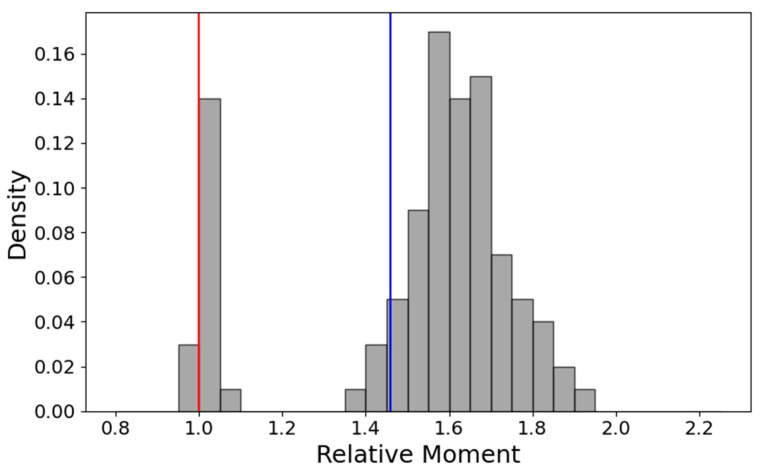
The histogram of I_af2ac_/I_model_ ratio for the NBD-LRR region. The blue line corresponds to the experimental inactive state, and the red line to the experimental active state.

The conformational preference toward more compact modeling solutions may be gauged in several ways. For instance, this results from the ratio of two principal moments—that of the presumably most extended model, the AF2—active control—used as an internal reference and that of a current model, I_af2ac_/I_model_. If the current model is less prolate, this ratio will be larger than 1. For each of the 32 CNLs, this ratio was measured for all AF2, AF3, RFAA and OmegaFold models. The histogram of this ratio for the NBD-LRR1-5 region—Figure 8 below—shows that over 80% of the models have this region in the more compact inactive ADP configuration.

**Figure 9:**
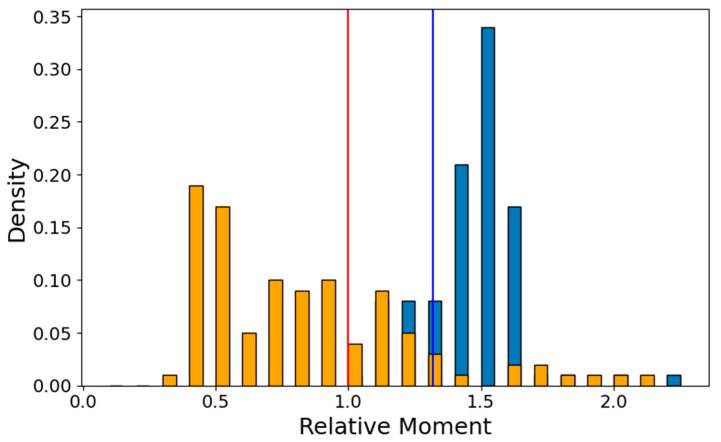
The histogram of I_af2ac_/I_model_ ratio for the CC-NBD-LRR1-5 region. The EDVID type is shown in blue bars, and the RPS5 type is shown in orange bars. The blue line corresponds to the experimental inactive state, and the red line to the experimental active state.

Given that half of the CNLs are RPS5-type helpers in which the CC does not interface anymore with the rest of the receptor and adopts random configurations (Figure 7)—the histogram for the overall CC-NBD-LRR1-5 is less well separated, as most of the helper models have the principal moment larger than that of their reference constrained to adopt an active ATP ZAR1-like configuration (Figure 9).

**Figure 10:**
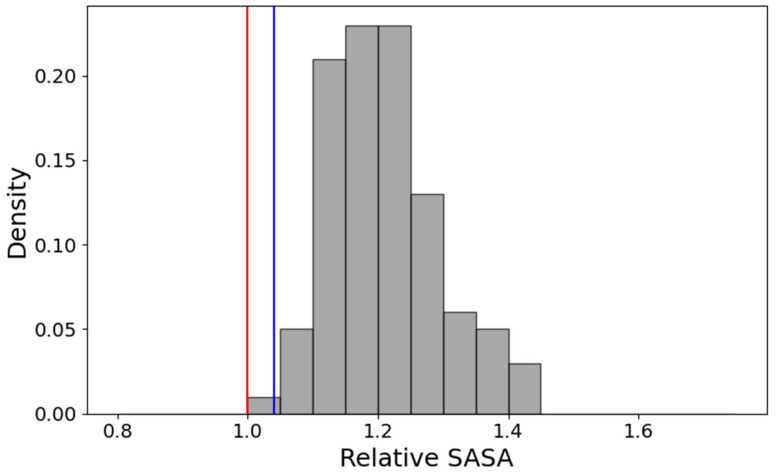
The histogram of ACC_af2ac_/ACC_model_ ratios for the CC-NBD-LRR1-5 region. The blue line corresponds to the experimental inactive state, and the red line to the experimental active state.

Nevertheless, even in these conditions, the overall accessibility is lower than that shown by the control. The histogram of accessibility ratios between AF2 active control (af2ac) references and the rest of the models for each CNL analyzed herein is shown in Figure 10. This indicates that all model solutions are more compact than the reference, mainly due to more buried residues in the CC region, even when this is wobbling randomly apart from the NDB-LRR1-5 core.

All these results indicate that, at least in the case of CNL receptors, AI platforms have a strong tendency to select the experimental, closed, compact ADP structure as a template to the detriment of the more extended ATP one.

**Figure 11:**
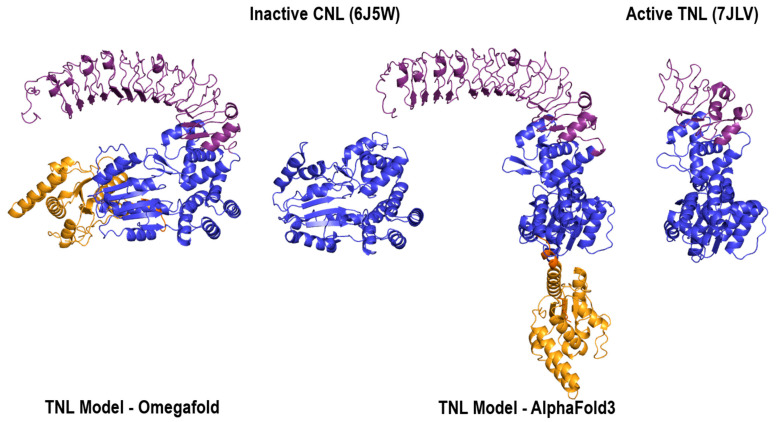
From left to right: Model of M1C2N4 (TNL) protein generated by OmegaFold with solved structure of NBS domain of ZAR1 protein; model of M1C2N4 generated by AlphaFold3 with solved structure of NBS and LRR domains of TNL protein ROQ1 (RCSB Code: 7JLV). For each structure: TIR domain—orange, NBS domain—blue, LRR domain: purple.

To assess what is happening when only one template is at hand, thankfully, a close CNL multidomain protein family does exist: the toll-interleukin-1 receptor (TIR) NOD-like receptors, TNL. In structural databases, only one entry is solved in the more extended ATP state. The modular organization of TNL mirrors that of CNL by swapping CC to TIR, which ends in a TIR/NBS-Arc1/Arc2/LRR four-module structure, which suffers the same conformational change of the central region upon activation as a CNL. This provided the opportunity to test one example of the influence of the template and MSA step in modeling the overall configuration of a multidomain multistate protein. Results on the TNL sequence M1C2N4 (retrieved from NLRscape) show that all MSA-based AI platforms—AF2, AF3 and RFAA—provide solutions following the active ATP template. In contrast, OmegaFold, which does not rely on MSA but rather on a protein language model based on a geometry-inspired transformer, models the TNL in a compact ADP conformation, as shown in Figure 11.

## 3. Discussion

### Model Quality

The recent release of several NOD-like receptor structures, including one present in the *A. thaliana* CNL set taken under investigation here, allowed a detailed analysis of the prediction capabilities of several AlphaFold and RoseTTAFold AI modeling platforms.

At the domain level, the structure of the folded NBD-Arc1, Arc2 and LRR modules belonging to CATH 3.40.50.300, 1.10.533.10 and 3.80.10 topologies have been predicted within less than 2 Å by all assessed platforms. In contrast, deviation from the experimental structure higher than 12 Å is seen in the coiled-coil N-terminal region. Analysis of the local solution provided by AI platforms for this region indicates that they combine structural information from the ADP-inactive and ATP-active templates of ZAR1. There are two key factors concurring with this issue: the first, more general, is the problem of multivalued 1D to 3D mappings, as is the case of all proteins solved in more than one structural state; the second, more specific, is the structural promiscuity and multivalency of CC domains. While mostly helical in terms of secondary structure, in plants, the CNL-CC region is known to adopt multiple, condition-dependent configurations and is shown to be able to stand alone or generate homo or hetero-multimers [23,29,49]. This highly raises the level of modeling inaccuracy, so departures from experiments are expected, and to pin down a closer-to-reality, context-dependent CC modeling solution, any kind of experimental constraints are of great help.

At the lower secondary structure level, detailed analysis carried out on the LRR domain indicates that despite the fact that the overall architecture is well predicted, mainly due to a consistent identification of ventral beta strands, there is a noticeable tendency on the dorsal side of the horseshoe to provide local helical solutions in experimentally coiled regions, most likely due to an excess of animal LRR templates in the default structural training set and/or a tendency of AI platforms toward providing local ordered more compact solutions. Hence, a careful review of the models provided by AI platforms for LRR domains, at least in the case of irregular plant NLR, is needed, as AI modeling platforms were shown to provide inconsistent local solutions [50] given their underrepresentation in structural databases and significant LRR motif irregularities [51].

At the global CNL architecture level, predictions based on the overall structural information used by default by AI platforms are all biased toward the more compact ADP-inactive configuration and only by feeding the AF2 predictor with filtered information specific to the ATP-active state this became apt to drive modeling toward the sparser ATP-active configuration. Notably, when the protein is modeled in the presence of ATP, the AF3 and RFAA platforms model the protein in its more compact ADP-inactive state, indicating that information related to ligand specificity is not taken into account in modeling the protein moiety but merely to place it at the right binding site. From an information point of view, this is surprising, as it would have been a very simple way to distinguish, in multistate situations, between active and inactive NBD templates, but seemingly, this was not used in the AF3 and RFAA network training workflows. Using ligands for template selection in such MSA-based AI platforms would be very useful in modeling the various states of proteins involved in signal transduction or proteins subjected to allostery.

The bias toward the more compact ADP-inactive state is, to a lesser extent, maintained in modeling all sequences on the ‘EDVID’ branch of *A. thaliana* CNLs. However, some sequences are modeled closer to the ATP-active state, suggesting that information on contacts at various interfaces of the CNL is missing from what was used by default in training the predictor. Surprisingly, the NeuroSnap implementation of RFAA was shown, in some cases, to be prone to generate entangled, overly compact models displaying severe steric clashes.

On the other hand, the sensitivity of AI platforms to the local, detailed characteristics of the sequence of a modular protein is plainly demonstrated by the vastly different modeling solutions proposed for the CNL receptors found on the ‘RPS5’ branch. All models of this group display the CC region as completely separated from the rest of the CNL. This solution correlates well with the presence of a myristoilation signal at the N-terminal end of the CNL sequence, which suggests a direct binding of the CNL to the membrane, in stark contrast to the insertion membrane mechanism that was proven for the EDVID-type receptor ZAR1. Even when separated from the NBD-LRR1-5 core, the overall exposed surface of the CC-NBD-LRR1-5 region is lower than that of the internal reference af2ac models, as seen in Figure 9. This adds up to the observation of a general trend toward more compact AI modeling solutions. This trend is also confirmed by several examples of CNLs from receptor families found in other plant species, which also show that when facing a choice between ADP compact and ATP-extended NBD templates, all AI platforms display a clear preference to model CNLs in the more compact state.

Interestingly, on the other hand, templates and the MSA step lead the way in finding AF2, AF3 and RFAA solutions. This is clearly shown by the TNL modeling example presented herein. All AF/RF default solutions provided for this receptor are models following the only existing template, which is in ATP extended TNL conformation. In contrast, in the solution provided by OmegaFold, which is not based on MSA, the receptor is modeled in an ADP compact state of the NBD, as shown in Figure 11. When feeding AF2 with only ADP templates and removing the MSA step, the resulting modeling solutions become inconsistent and diverge from both the NBD (NBS-Arc1/Arc2) standard, well-documented states.

It is generally recognized that modeling multidomain proteins, especially those that undergo structural transitions during their functional cycle, is far more complex than modeling single-domain proteins [1], and the present case study plainly shows many of the confronting challenges. For multidomain multistate proteins, AI platforms were reported to predict only a single ‘ground state’ [52]. Here, however, 20% of the selected CNL sets were not modeled in the ground but instead in the active snapshot, probably due to the lack of information regarding interdomain contacts. Another surprise is that simple information, such as the nature of the ligand, is not used by the network to discriminate between protein states by the new generation of predictors. Our AF2 inactive/active MSA models show that such an approach would be well suited to discriminate between states. The low accessibility of the RPS5-type helpers is also notable. This suggests that the local solution of the CC is more compact than that of the elongated one seen in active ZAR1. This is consistent with previous reports that AIs do not perform very well in modeling elongated CC regions, which are modeled in some cases as ‘balls’ [53].

## 4. Materials and Methods

### 4.1. Sequence Selection of the A. thaliana Representative CNL Set

A representative set of *A. thaliana* CNL sequences was selected from the NLRscape web resource (https://nlrscape.biochim.ro/home.php, accessed on 20 May 2024). In the first step, only sequences containing the canonical CC-NBD-LRR domain organization were retained, while those containing additional sequence stretches at any of the N- and C-terminus ends were eliminated. The retained set was further filtered out using NLRexpress (https://nlrexpress.biochim.ro/, accessed on 20 May 2024) to discard sequences lacking one or more of the 9 characteristic CNL motifs while retaining only the fully functional predicted ones. This reduced set was then clustered using MMSeqs2 at 70% coverage and 70% identity. Cluster representatives were finally selected based on the level of literature mentioned and the degree of experimental usage.

### 4.2. Sequence Analysis of Representative Set Sequences

Relevant data related to representatives and their structural profiles were retrieved from NLRscape, while the delineation of the CC, NBD and LRR canonical domains and of the two invariant globular NBD regions, NBS-Arc1 and Arc2, was based on sequence profiling and the precise location of the 9 CNL motifs identified with NLRexpress and LRRpredictor.

The phylogenetic analysis was conducted using the maximum likelihood method implemented in IQtree [54] (http://iqtree.cibiv.univie.ac.at/, accessed on 20 August 2024). This analysis was performed on either the full-length sequences or individual domains and subdomains. Sequence alignments were performed using MAFFT v7.490 [55]. The phylogeny model was selected using the automated substitution selection and FreeRate model to account for heterogeneity [56]. Additionally, we employed the ultrafast bootstrap approximation [57] and the SH-aLRT branch test, both utilizing 1000 replicates, alongside the approximate Bayes test [58]. Tree graphics were generated using iTOL v7.0 (https://itol.embl.de/, accessed on 20 August 2024) [59]. Sequence identity matrices were computed on either the full-length sequences or individual domains and subdomains using Ugene v46.0 [60] and in-house scripts.

### 4.3. Template and MSA Filtering with the Locally Implemented Version of AF2

In order to assess the effects induced by template and MSA restriction/enrichment on AI modeling, the latest version of AlphaFold2 (v. 2.3.2) was locally implemented and used to test various retraining filtering.

Firstly, since the generation of the PDB snapshot that is used by default by AF2, which already contains the *A. thaliana* ZAR1 CNL structures solved in active ATP open conformation (6J5T), transition conformation and inactive ADP closed conformation (6J5W), newer structures of NLR proteins have been published (8RFH, 8XUO, 8XUQ, 8XUV) [61,62]. In order to test the effects of template restrictions in guiding AF2, these new templates were split into “Active” and “Inactive” conformations based on their ligand/structural similarity to ZAR1, as presented in Table 6. Other plant proteins that were used are also presented.

Secondly, as the CC and LRR domains have well-described architectures, Cath 1.20 Up-Down Bundle and Cath 3.80.10 Leucine-Rich Repeat, respectively, the AF2 PDB database was filtered out to retain only protein chains containing regions of these two types. Subsequently, only these were included as input in the final modeling of the “MSA” models.

Moreover, given that the MSA that AF2 generates should reflect features specific to NLR proteins, input sequences were filtered out to retain only the complete set of plant NLRs retrieved from NLRScape.

Given that the structure of *A. thaliana* ZAR1 was already solved in its inactive, transition and active states, its default and restricted template models were used to gauge the prediction capabilities of the AI platforms tested herein. In order to improve the local modeling of ZAR1 (Q38834), the lab-implemented version of AF2 was modified to accept templates with higher than 95% sequence similarity to the input sequence.

Files containing all sequence names and all PDB accession codes for the structures used are available on the above-mentioned GitHub Repository.

### 4.4. Model Generation

Default AF2 models were retrieved from the AlphaFold2 Database [63]. AlphaFold2 v. 2.3.2, downloaded from the Deepmind Github repository [64], was used for local implementation. The installed AF2 has been run on consumer-grade desktops equipped with NVIDIA RTX 3080Ti GPUs. Instead of a docker deployment, AF2 was directly installed, given that this allows a more simple and flexible handling. AF2 databases were modified to pass the customized sequences/structures subsets as described above. In this way, 4 model sets were generated using the local AF2 deployment:

- No MSA input, only the “Active” experimental structures from Table 6—“Active Control”.
- No MSA input, only the “Inactive” experimental structures from Table 6—“Inactive Control”.
- MSA consisting of only NLR Proteins retrieved from NLRscape, “Active” experimental structures from Table 6 and structures corresponding to CATH families for CC and LRR architectures—“Active MSA”.
- MSA consisting of only NLR Proteins retrieved from NLRscape, “Inactive” experimental structures from Table 6 and structures corresponding to CATH families for CC and LRR architectures—“Inactive MSA”.

To test the effect of ligand presence upon CNL protein modeling, AlphaFold3 and the NeuroSnap server implementation of RoseTTAFold All Atom RFAA were used to model all sequences in interaction or not with their endogenous ADP/ATP ligands. Hence, all sequences of the representative set were modeled on these two web platforms in: (a) the presence of ADP, corresponding to the active state; (b) the absence of ligands, corresponding to the transition state; and (c) the presence of ATP, corresponding to the active state.

In this way, for each sequence of the representative set, overall, 39 models were generated as follows: 1 AF2-DB, 3 NeuroSnap-RFAA3, 4 × 5 AF2 local and 3 × 5 AF3 models ranked from 1 to 5 in these later cases. All of these were further subjected to refinement and analysis, as described below.

For the conformation preference analysis (Section 2.2.4), as a control to the template and MSA-based AF/RFAA predictors, we also analyzed the models generated by OmegaFold, which relies on a protein language model based on a geometry-inspired transformer rather than MSA [65].

### 4.5. Model Refinement

Raw models were first scored with MolProbity v4.3. All models were then optimized by energy minimization using the L-BFGS algorithm implemented in OpenMM v. 7.3.0 and then rescored. For RFAA models, a further simulated annealing was needed. This was performed by heating the models to 600 K in 0.6 ns followed by a 1 ns MD simulation and a stepwise cooling from 600 K to 300 K in 1 ns, from 300 K to 100 K in 2 ns and from 100 K to 0 K in 3 ns, with final L-BFGS minimization. In order to preserve the secondary structures, harmonic constraints were applied on backbone atoms in these regions, using a 10 kcal/nm^2^ force constant. In the end, MolProbity scores of RFAA models for the resulting final state were obtained.

Simulation files were generated using the tLEaP from the AmberTools23 package [66]. Models were parameterized using the FF19SB forcefield [67], and parameters for ATP/ADP ligands were taken from Meagher et al. [68].

### 4.6. Model Analysis

#### 4.6.1. Model Quality

The quality of all models was evaluated using MolProbity. Moreover, interdomain distances between all heavy atoms were used to probe for unrealistic clashes (<2 Å) among the CC, NBD and LRR domains. These criteria were also used in the optimization protocol described above.

#### 4.6.2. Assessment of Overall Architecture

The RCSB PDB database contains NLR structures solved in multiple states: ADP-inactive monomer, transition and ATP-active oligomers. These differ mainly by the configuration of the NBD ‘switch’, in that Arc2 performs a ~180° rotation with respect to NBS-Arc1 during activation. In a simplified representation, this can be seen as a flip of the Arc2 mass center (m.c.) to the opposite side of the plan formed within NBS-Arc1 by the C_α_ of the conserved Gly from the entry NDB motif: VVG, the m.c. of NBS and the m.c. of Arc1. In comparing the module architecture of various CNL models, this plan with the origin in the entry Gly C_α_ was used to represent the relative location of Arc2 m.c., which allows to evaluate if the model is in ADP-closed or ATP-open state, but also to locate the m.c. of the CC and LRR1-5 to see if the overall architecture is consistent or diverges from that of ZAR1—the only experimentally known CNL structure. Given that the number of LRR repeats largely varies over the representative set, only the first 5 repeats known to interact with NBD in the ADP closed state [25] were considered in the m.c. calculation of LRR (LRR1-5).

Principal moments and SASA were calculated using the respective functions from the MDTraj package [69].

#### 4.6.3. Model Stability

Model stability was tested by two simulation methods. For the generated ZAR1 models, a more detailed approach was used to compare structural prediction performance. The top models in each class, as well as the crystallized structures, were immersed into an explicit water box, and MD was simulated using OpenMM at 300 K° and constant pressure for 10 ns. Since measuring interdomain interface interactions was of special interest, SHAKE use was avoided, opting instead for 1 fs timestep simulation, allowing free hydrogen movements.

Secondly, the stability of all generated models was assessed by a higher sampling efficacy method, namely the generalized coordinate Hamiltonian Monte Carlo (GCHMC) [70], implemented in the Robosample package. The method is able to use constraints without disrupting the correct conformation probability distribution. As GCHMC uses both Cartesian and bond–angle–torsion coordinates, this ensures great freedom in choosing constraints. Herein, three groups of degrees of freedom (Gibbs blocks) took turns to oversample the interfaces between the three domains and the linker areas: one sampling all of the internal degrees of freedom (DoFs) of the system, one for sampling the torsional DoFs on the interface residues (defined as residues that are closer than 5A to residues on other domains) and one for sampling the interface sidechains and the phi/psi torsions of the linker domains at the same time. All simulation parameters are provided on the above-mentioned GitHub repository.

#### 4.6.4. Interface Analysis

Since NLR proteins are multidomain, it is important to evaluate how well the generated models conserve the interfaces between the domains as well as how stable the interfaces are in time. To this end, using the above-described molecular dynamics trajectories, we employed Prodigy [71] to compute the binding free energy between each pair of domains (CC-NBD, CC-LRR and NBD-LRR) for a subset of snapshots equally spaced across each simulation.

## 5. Conclusions

The present work focuses on evaluating the ability of some of the best-known platforms of new-generation deep learning predictors to provide plausible models in the more challenging situation of multistate multidomain proteins by taking as a case study a coiled-coil family of NOD-like receptors from *A. thaliana*. This class is interesting on at least two fronts: First—some structural information in this group does exist, but it is not comprehensive, which allows both gauging the AI predictor performance and evaluating extrapolation effects induced by sequence drifts when the overall modular architecture is conserved. Second—an in-depth understanding of the functioning of this immune system family has a tangible economic impact on plant yield [72]; thus, any structural information might be of use in this case—this is why this work was directed toward the most frequently used CNL receptors in experiments.

Results presented herein reveal a truly remarkable ability of these platforms to correctly predict the 3D structure of the sequence modules that fold in well-established topologies. On the bright side, there is also the sensitivity of these platforms to local sequence drifts upon the modeling solution proposed for the overall modular configuration of the multidomain protein—as shown in the present work by the structural discrimination between the two main CNL branches. As expected, on the other hand, one can notice their lower performance in modeling more promiscuous, morphing regions of a modular sequence, such as the coiled-coil regions, which locally display strong helical propensity and may be found in multiple global, context-dependent configurations. In such cases, feeding the predictor with filtered structural information rather than with the default, a general one, proved useful in directing the modeling process closer to reality. Results also indicate that in multivalued 1D to 3D instances, when several templates are at hand for the same sequence, AI predictors display a consistent preference to model such modular proteins in their most compact configuration rather than in the sparser ones. A similar bias was noticed at the local secondary structure level as well—for instance, in modeling many dorsal coiled stretches of the LRR domain as helices.

The trends revealed by this work suggest that using the new generation of AI predictors for modeling multidomain multistate proteins is fruitful when global templates are at hand, but nevertheless, this approach faces the challenges mentioned above that have to be taken into account. Challenges further increase when global templates are missing and only local remote homologs may be found in structural databases. In this case, in our hands, a piecewise modeling approach using such predictors, with local AI models further corrected for large insertions and joined to include all known experimental constraints such as those given by SAXS [73], Cryo-EM [74], NMR [75] or HDX-MS [76] in building the overall modular architecture is better suited to give results closer to the real functional system.

Finally, deep learning predictions are mostly based on the empiric evidence gathered through the ages in structural databases; it is highly relevant to explore the properties of such information-based models with methods focused on the physics of the modular system, such as structural optimization, stability tests and/or probing their potential energy surface (PES), in order to identify local barriers and configurational basins, especially when interactions with ligands or other molecular species are known to shape up the modular architecture. While MD simulation provides a detailed description of the system, The new generation of enhanced sampling methods, such as GCHMC, are shown to probe a far larger conformational space of the system and, in this sense, should be more useful, for instance, in probing the pathways between states of multistate multidomain proteins.

## Figures and Tables

**Table 1:** RMSD (Å) of individual domains for the highest ranked models using an active ATP state structure as reference.

Model Name	CC	NBS-ARC1	ARC2	LRR
AF2—Database	14.46	1.86	1.63	1.45
AF2—Active Control	1.39	0.49	0.42	0.53
AF2—Inactive Control	18.51	1.86	1.51	0.84
AF2—Active MSA	1.55	0.59	0.61	0.96
AF2—Inactive MSA	14.40	1.79	1.53	0.97
AF3—ADP	14.41	1.80	1.64	1.42
AF3—ATP	14.39	1.89	1.58	1.33
AF3—No Ligand	14.45	1.92	1.59	1.51
RFAA—ADP	14.42	2.04	1.64	1.86
RFAA—ATP	14.50	2.05	1.78	2.02
RFAA—No Ligand	14.45	1.92	1.65	1.96
OmegaFold	14.48	1.87	1.33	3.11

**Table 2:** RMSD (Å) of individual domains for the highest ranked models using the Inactive ADP state structure as reference.

Model Name	CC	NBS-ARC1	ARC2	LRR
AF2—Database	12.42	1.33	0.88	1.34
AF2—Active Control	19.19	1.89	1.45	0.78
AF2—Inactive Control	0.80	0.69	0.35	0.47
AF2—Active MSA	18.48	1.88	1.51	0.90
AF2—Inactive MSA	12.54	0.86	0.44	0.75
AF3—ADP	11.99	1.31	0.98	1.22
AF3—ATP	12.11	1.35	0.87	1.16
AF3—No Ligand	12.08	1.26	0.93	1.37
RFAA—ADP	12.74	1.41	1.02	1.77
RFAA—ATP	12.65	1.53	1.04	1.92
RFAA—No Ligand	12.36	1.42	1.11	1.89
OmegaFold	12.99	1.24	0.87	3.04

**Table 3:** Global RMSD (Å) for the highest ranked models using crystal structures as reference.

Model Name	RMSD vs. Active	RMSD vs. Inactive
AF2—Database	22.158	6.004
AF2—Active Control	0.832	23.036
AF2—Inactive Control	22.612	0.675
AF2—Active MSA	1.271	22.873
AF2—Inactive MSA	21.997	5.837
AF3—ADP	22.119	5.785
AF3—ATP	22.076	5.879
AF3—No Ligand	22.191	5.889
RFAA—ADP	22.406	6.756
RFAA—ATP	22.646	6.792
RFAA—No Ligand	22.424	6.027
OmegaFold	22.374	6.442

**Table 4:** Binding free energy between ZAR1 domains. Energies reported in kcal/mole.

Model Name	CC/NBD Interface	NBD/LRR Interface	CC/LRR Interface
Inactive Crystal	−8.8	−14.0	−7.8
AF2—Database	−10.1	−15.1	−8.5
AF2—Inactive Control	−9.7	−14.7	−8.3
AF2—Inactive MSA	−10.1	−14.9	−8.6
AF3—ADP	−10.9	−15.6	−8.9
AF3—ATP	−10.7	−15.0	−9.2
AF3—No Ligand	−10.6	−15.4	−9.1
RFAA—ADP	−9.7	−15.0	−9.6
RFAA—ATP	−9.7	−16.2	−9.3
RFAA—No Ligand	−9.7	−14.4	−9.2

**Table 5:** The 36 CNL clusters represented by the member with the most publications in each cluster. Clusters that were eliminated are written in bold.

Protein Name	Length	Cluster	Domains	Publications
A0A1P8AP86	888	1	CC-NBD-ARC1-ARC2-LRR	2
Q9SI85	893	2	CC-NBD-ARC1-ARC2-LRR	3
Q940K0	889	3	CC-NBD-ARC1-ARC2-LRR	6
Q9M667	835	4	CC-NBD-ARC1-ARC2-LRR	5
Q9C646	899	5	CC-NBD-ARC1-ARC2-LRR	3
Q39214	926	6	CC-NBD-ARC1-ARC2-LRR	10
Q8W474	907	7	CC-NBD-ARC1-ARC2-LRR-X	4
Q8RXS5	888	8	CC-NBD-ARC1-ARC2-LRR	3
Q8W3K3	910	9	CC-NBD-ARC1-ARC2-LRR	3
O64973	889	10	CC-NBD-ARC1-ARC2-LRR	10
A0A654EJG6	904	11	CC-NBD-ARC1-ARC2-LRR	0
Q8L3R3	885	12	CC-NBD-ARC1-ARC2-LRR	4
Q8W4J9	908	13	CC-NBD-ARC1-ARC2-LRR	12
Q9STE5	847	14	CC-NBD-ARC1-ARC2-LRR	2
Q9LQ54	870	15	CC-NBD-ARC1-ARC2-LRR	3
**A0A7G2ET34**	**1306**	**16**	**X-CC-NBD-ARC1-ARC2-LRR**	**0**
Q9XIF0	906	17	CC-NBD-ARC1-ARC2-LRR	2
Q9LVT3	948	18	CC-NBD-ARC1-ARC2-LRR-X	2
Q9STE7	847	19	CC-NBD-ARC1-ARC2-LRR	2
Q9FG90	862	20	CC-NBD-ARC1-ARC2-LRR	2
**Q9LRR5**	**1424**	**21**	**CC-NBD-ARC1-ARC2-LRR-X-LRR**	**2**
Q9FLB4	874	22	CC-NBD-ARC1-ARC2-LRR	2
A0A5S9WIX4	875	23	CC-NBD-ARC1-ARC2-LRR	0
**A0A5S9WPD4**	**1025**	**24**	**CC-NBD-ARC1-ARC2-LRR-X**	**0**
**A0A654EJV2**	**661**	**25**	**CC-NBD-ARC1-ARC2-LRR**	**0**
A0A654FPA2	881	26	CC-NBD-ARC1-ARC2-LRR	0
O82484	892	27	CC-NBD-ARC1-ARC2-LRR	2
Q8W3K0	1138	28	CC-NBD-ARC1-ARC2-LRR	5
Q38834	852	29	CC-NBD-ARC1-ARC2-LRR	10
Q9LRR4	1054	30	CC-NBD-ARC1-ARC2-LRR	2
P60839	884	31	CC-NBD-ARC1-ARC2-LRR	2
P60838	894	32	CC-NBD-ARC1-ARC2-LRR	5
A0A654EJC3	921	33	CC-NBD-ARC1-ARC2-LRR	0
Q9LMP6	851	34	CC-NBD-ARC1-ARC2-LRR	2
Q9SX38	857	35	CC-NBD-ARC1-ARC2-LRR	2
Q42484	909	36	CC-NBD-ARC1-ARC2-LRR	17

**Table 6:** Table containing relevant solved structures of NLR proteins. The ZAR1 structures have been bolded.

UniProt Acc.	RCSB Acc	Chain	Domains	Organism	Geometric Classification
Q38834	6J5T	C	CC-NBD-ARC1-ARC2-LRR	*A. thaliana*	Active
Q38834	6J6I	C	CC-NBD-ARC1-ARC2-LRR	*A. thaliana*	Active
Q9ZSN5	7CRC	A	X-TIR-NBD-ARC1-ARC2-LRR	*A. thaliana*	Active
Q9ZSN5	7DFV	A	X-TIR-NBD-ARC1-ARC2-LRR	*A. thaliana*	Active
A0A290U7C4	7JLV	A	TIR-NBD-ARC1-ARC2-LRR-X	*N. benthamiana*	Active
S5ABD6	7XC2	A	CC-NBD-ARC1-ARC2-LRR	*T. monococcum*	Active
S5ABD6	7XE0	A	CC-NBD-ARC1-ARC2-LRR	*T. monococcum*	Active
S5ABD6	7XX2	A	CC-NBD-ARC1-ARC2-LRR	*T. monococcum*	Active
Q38834	6J5W	A	CC-NBD-ARC1-ARC2-LRR	*A. thaliana*	Inactive
A1X877	6S2P	N	CC-NBD-ARC1-ARC2-LRR	*S. lycopersicum*	Inactive
A1X877	8BV0	A	CC-NBD-ARC1-ARC2-LRR	*S. lycopersicum*	Inactive
A0A0S3ANR1	8RFH	A	CC-NBD-ARC1-ARC2-LRR	*N. benthamiana*	Inactive
A0A3Q7IF17	8XUO	A	CC-NBD-ARC1-ARC2-LRR	*S. lycopersicum*	Inactive
A0A3Q7IF17	8XUQ	A	CC-NBD-ARC1-ARC2-LRR	*S. lycopersicum*	Inactive

## Data Availability

Data is contained within the article and Supplementary Materials, at the presented GitHub URL.

## Supplementary Materials

The Supplementary Figure S1 and Tables S1–S5 can be downloaded at: https://www.mdpi.com/article/10.3390/ijms26020500/s1. All generated models and files containing filtered input for AlphaFold2 can be downloaded at: https://github.com/Teohoho/CNL_Paper_Data.
