# Supplementary material for: Lessons from Deep Learning Structural Prediction of Multistate Multidomain Proteins—The Case Study of Coiled-Coil NOD-like Receptors
Source: Int J Mol Sci. 2025 Jan 9;26(2):500. doi: 10.3390/ijms26020500 (PMC11765006; doi:10.3390/ijms26020500)
Supplement: Supplementary file 1 [file ijms-26-00500-s001.zip › ijms-3367898-supplementary.pdf]

Figure S1 – CNL sequence alignment. Visualization done in Ugene Software, cited in main text.

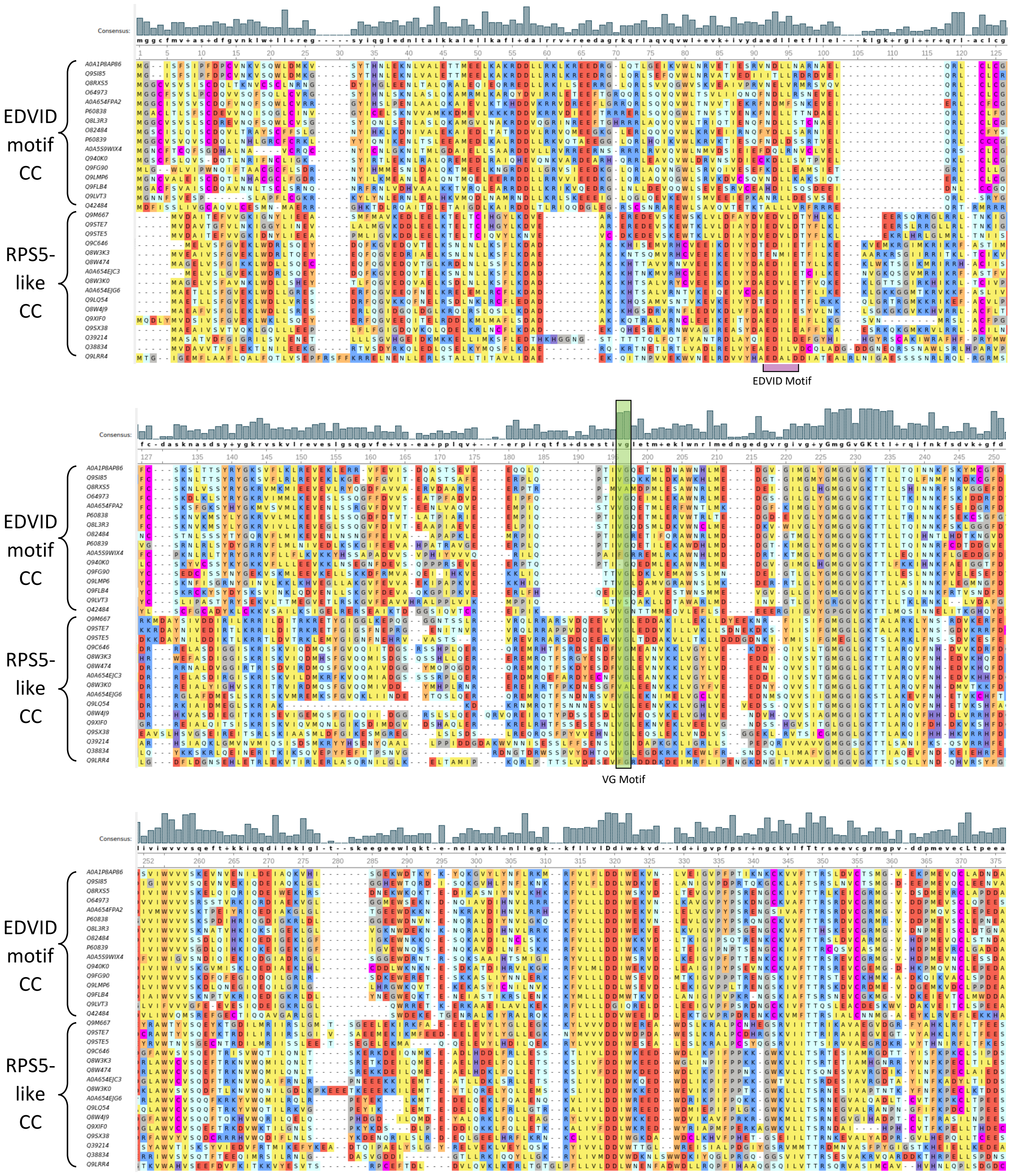

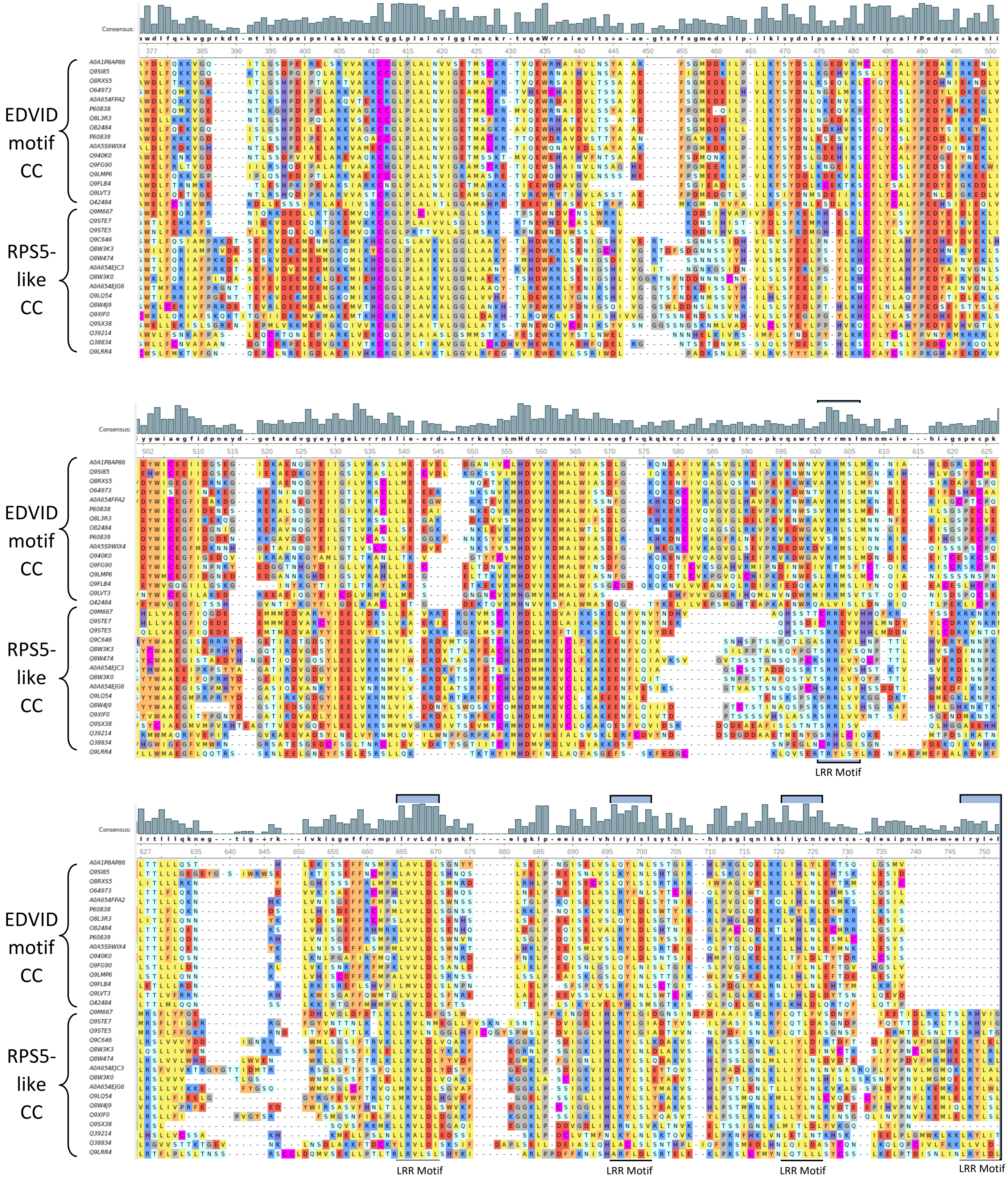
Consensus:
EDVID motif CC
RPS5-like CC
LRR Motif
LRR Motif
LRR Motif
LRR Motif
LRR Motif

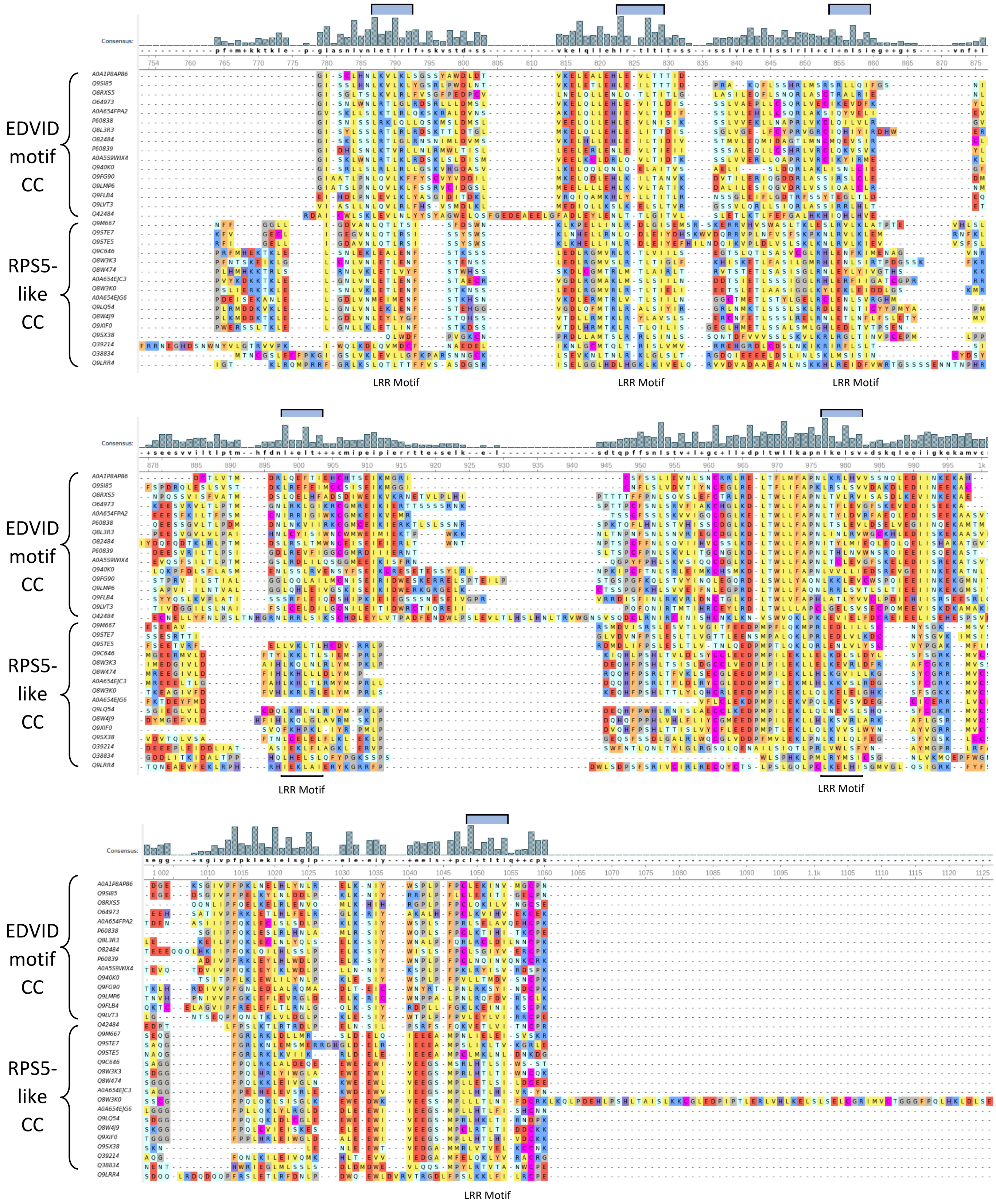
Consensus:
EDVID motif CC
RPS5-like CC
LRR Motif
LRR Motif
LRR Motif
Consensus:
EDVID motif CC
RPS5-like CC
LRR Motif
LRR Motif
Consensus:
EDVID motif CC
RPS5-like CC
LRR Motif

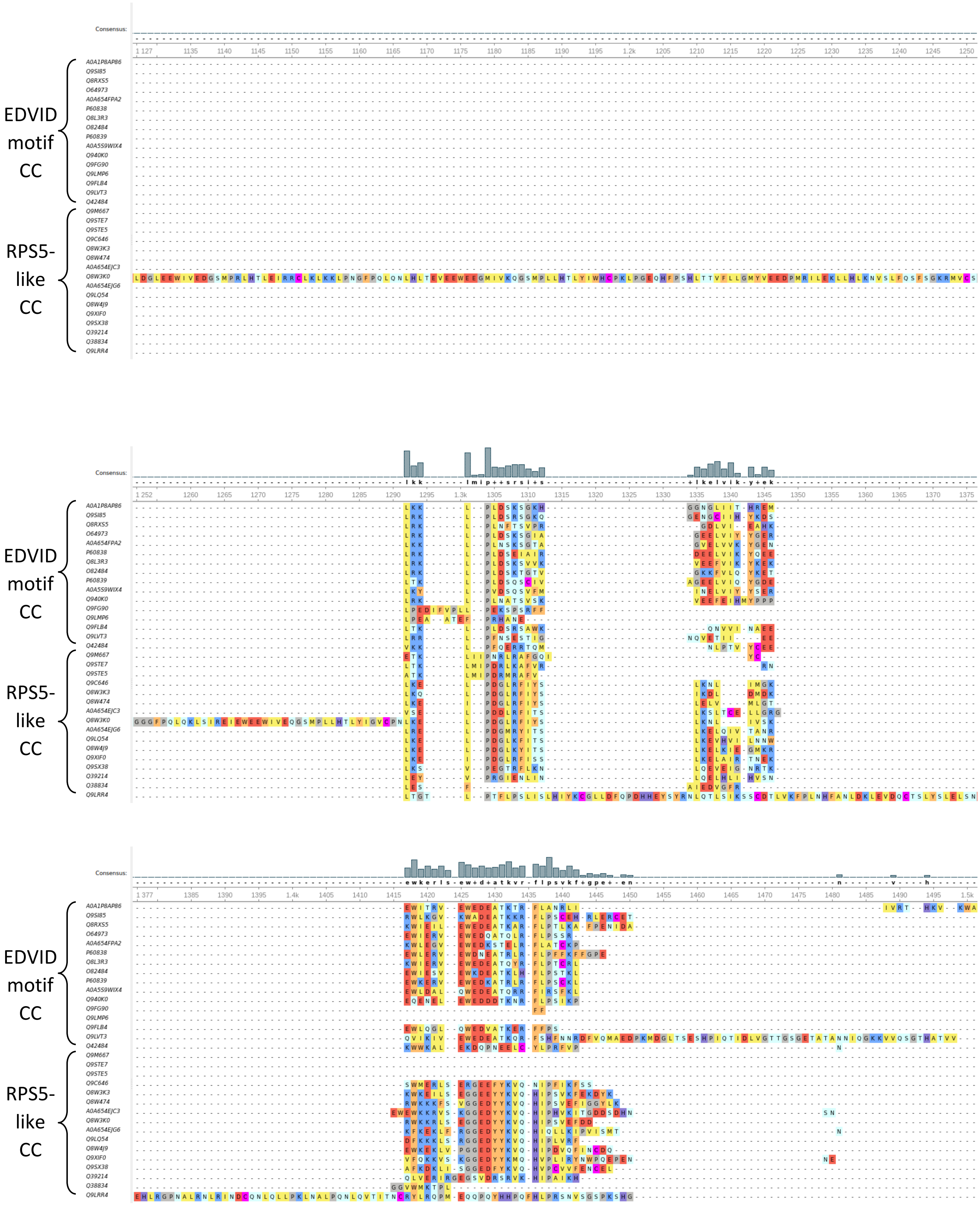
Consensus:
EDVID motif CC
RPS5-like CC
A0A1P8AP86
Q9SI85
Q8RXS5
O64973
A0A654FPA2
P60838
Q8L3R3
O82484
P60839
A0A5S9WIX4
Q940K0
Q9FG90
Q9LMP6
Q9FLB4
Q9LVT3
Q42484
Q9M667
Q9STE7
Q9STE5
Q9C646
Q8W3K3
Q8W474
A0A654EJC3
Q8W3K0
A0A654EJG6
Q9LQ54
Q8W4J9
Q9XIF0
Q9SX38
Q39214
Q38834
Q9LRR4

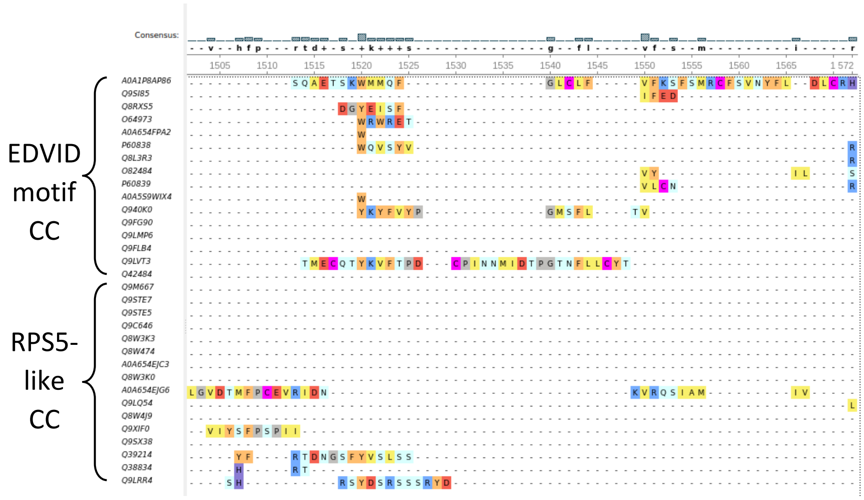
Consensus:
EDVID motif CC
RPS5-like CC
A0A1P8AP86
Q9SI85
Q8RXS5
O64973
A0A654FPA2
P60838
Q8L3R3
O82484
P60839
A0A5S9WIX4
Q940K0
Q9FG90
Q9LMP6
Q9FLB4
Q9LVT3
Q42484
Q9M667
Q9STE7
Q9STE5
Q9C646
Q8W3K3
Q8W474
A0A654EJC3
Q8W3K0
A0A654EJG6
Q9LQ54
Q8W4J9
Q9XIF0
Q9SX38
Q39214
Q38834
Q9LRR4

Table S1 – MolProbity scores before geometry optimization

	AF2 DB	AF2 AC					AF2 IC					AF2 AM					AF2 IM					AF3 - No Ligand					AF3 - ADP					AF3 - ATP					RFAA - No Ligand	RFAA - ADP	RFAA - ATP
Sequence	Rank 0	Rank 0	Rank 1	Rank 2	Rank 3	Rank 4	Rank 0	Rank 1	Rank 2	Rank 3	Rank 4	Rank 0	Rank 1	Rank 2	Rank 3	Rank 4	Rank 0	Rank 1	Rank 2	Rank 3	Rank 4	Rank 0	Rank 1	Rank 2	Rank 3	Rank 4	Rank 0	Rank 1	Rank 2	Rank 3	Rank 4	Rank 0	Rank 1	Rank 2	Rank 3	Rank 4	Rank 0	Rank 0	Rank 0
Q9LVT3	1.785	3.570	3.585	3.490	3.489	3.685	3.554	3.511	3.491	3.555	3.657	2.861	2.808	2.819	3.025	2.789	2.898	2.691	3.024	2.770	2.856	2.520	2.537	2.520	2.502	2.512	3.090	2.982	2.941	2.763	3.062	3.088	3.037	3.049	3.144	3.099	3.305	3.265	3.309
A0A654FPA2	1.010	3.174	3.301	3.498	3.503	3.710	3.291	3.297	3.544	3.542	4.191	2.126	2.143	2.262	2.276	2.127	2.091	2.183	2.107	2.176	2.187	2.452	2.518	2.482	2.414	2.486	2.481	2.413	2.529	2.603	2.617	2.510	2.546	2.636	2.517	2.517	2.844	2.772	3.012
Q9M667	1.387	3.289	3.359	3.509	3.613	4.159	2.742	2.750	3.504	3.531	4.294	2.434	2.432	2.760	2.600	2.732	2.767	2.427	2.510	2.492	2.579	2.693	2.731	2.674	2.695	2.731	2.825	2.786	2.751	2.760	2.835	2.732	2.769	2.778	2.841	2.915	2.902	2.964	3.008
A0A654EJC3	1.376	3.238	3.244	3.558	3.462	3.809	2.966	2.627	3.508	3.458	4.079	2.619	2.674	2.670	2.498	2.664	2.665	2.739	2.615	2.611	2.685	2.603	2.621	2.552	2.575	2.581	2.898	2.816	2.831	2.819	2.778	3.039	2.800	2.820	2.866	3.059	2.942	2.953	3.061
A0A654EJG6	1.721	3.084	3.179	3.560	3.460	3.960	3.011	2.851	3.538	4.433	3.581	2.726	2.671	2.757	2.820	2.621	2.587	2.700	2.692	2.872	2.674	2.548	2.545	2.516	2.541	2.505	2.702	2.721	2.895	2.944	2.934	2.804	2.894	2.983	2.838	2.777	3.146	3.068	3.186
Q9FLB4	1.241	3.509	3.441	3.676	3.312	3.672	3.365	3.454	3.992	3.351	3.682	2.216	2.301	2.362	2.321	2.227	2.152	2.213	2.154	2.232	2.358	2.562	2.589	2.506	2.593	2.593	2.696	2.805	2.737	2.808	2.821	2.724	2.664	2.859	2.716	2.801	3.005	2.921	2.995
Q8W3K0	1.436	3.645	3.581	3.821	3.564	3.953	3.707	3.718	3.628	3.533	4.234	2.755	2.424	2.595	2.716	2.765	2.489	2.505	2.762	2.600	2.571	2.705	2.737	2.626	2.644	2.605	2.887	2.877	2.804	2.893	2.895	3.032	2.907	2.826	2.814	2.986	3.522	3.747	3.603
Q38834	1.227	2.258	2.230	3.542	3.365	3.641	2.267	2.373	3.458	3.484	3.657	2.260	2.487	2.231	2.256	2.192	2.243	2.451	2.253	2.212	2.235	2.454	2.482	2.454	2.499	2.477	2.628	2.724	2.697	2.557	2.818	2.803	2.880	2.622	2.690	2.792	2.716	2.796	2.911
Q9LQ54	1.441	3.118	3.144	3.524	3.552	3.619	2.930	2.941	3.590	3.548	3.649	2.575	2.447	2.589	2.842	2.548	2.680	2.586	2.608	2.648	2.555	2.675	2.888	2.742	2.711	2.716	2.908	3.285	3.145	2.994	2.995	3.059	3.030	3.114	3.084	3.013	3.053	3.270	3.066
Q9LRR4	1.385	3.325	3.468	3.799	3.540	3.990	3.333	3.411	3.703	3.521	3.904	2.751	2.700	2.837	2.700	2.827	2.617	2.770	2.615	2.727	2.825	2.575	2.566	2.604	2.577	2.577	2.875	2.885	2.685	2.779	3.101	2.732	2.820	2.850	2.832	2.827	3.240	3.116	3.180
Q9STE7	1.450	3.397	3.322	3.488	4.247	3.704	2.973	3.028	4.104	3.674	3.759	2.463	2.446	2.506	2.756	2.542	2.447	2.734	2.556	2.408	2.565	2.587	2.638	2.559	2.644	2.801	3.081	2.895	2.973	2.996	2.954	2.896	2.924	2.761	2.792	2.903	2.883	3.084	2.977
A0A5S9WPD4	1.162	3.651	3.525	3.538	3.458	4.164	3.555	3.511	3.789	3.458	3.831	2.521	2.607	2.620	2.726	2.434	2.412	2.725	2.568	2.907	2.559	2.503	2.435	2.427	2.397	2.397	2.963	2.950	2.884	2.807	2.922	2.977	2.862	2.877	2.803	2.793	3.670	3.647	3.925
Q8W4J9	1.321	3.028	3.080	3.534	3.690	3.740	2.845	3.022	3.986	3.539	3.711	2.541	2.351	2.415	2.503	2.570	2.449	2.388	2.266	2.540	2.511	2.513	2.517	2.582	2.542	2.486	2.755	2.666	2.748	2.843	2.881	2.691	2.628	2.657	2.749	2.775	2.849	3.187	3.158
O64973	1.197	3.259	3.394	3.595	3.592	3.696	3.309	3.411	3.507	3.612	3.690	2.454	2.333	2.540	2.339	2.316	2.260	2.336	2.274	2.268	2.458	2.474	2.499	2.541	2.478	2.439	2.714	2.653	2.704	2.636	2.727	2.658	2.835	2.777	2.885	2.674	2.946	3.255	2.970
Q9SI85	1.074	3.399	3.361	3.501	3.624	3.713	3.464	3.439	3.839	3.500	3.690	2.265	2.175	2.192	2.132	2.236	2.232	2.205	2.162	2.213	2.254	2.551	2.443	2.470	2.461	2.452	2.830	2.712	2.664	2.745	2.685	2.994	2.760	2.748	2.679	2.837	2.916	2.930	3.197
Q8L3R3	1.260	3.368	3.333	3.568	3.609	3.767	3.341	3.385	3.454	3.499	3.643	2.132	2.245	2.229	2.257	2.256	2.153	2.382	2.294	2.249	2.295	2.512	2.471	2.520	2.501	2.484	2.583	2.700	2.492	2.618	2.453	2.638	2.609	2.599	2.602	2.471	2.772	2.834	3.012
Q940K0	1.172	3.294	3.248	3.450	3.593	3.815	3.358	3.302	3.448	3.457	3.794	2.430	2.335	2.345	2.397	2.395	2.348	2.385	2.278	2.406	2.559	2.467	2.496	2.475	2.559	2.508	2.646	2.674	2.721	2.698	2.626	2.611	2.608	2.826	2.582	2.623	3.098	3.390	3.147
P60838	1.135	3.313	3.343	3.507	4.005	3.719	3.390	3.362	3.491	3.645	3.672	2.356	2.480	2.327	2.533	2.296	2.286	2.287	2.266	2.301	2.478	2.499	2.575	2.622	2.682	2.518	2.658	2.862	2.757	2.765	2.910	2.746	2.816	2.888	2.892	2.898	2.934	2.954	2.911
O82484	1.086	3.269	3.414	3.474	3.579	3.616	3.322	3.375	3.473	3.999	3.599	2.396	2.514	2.516	2.395	2.316	2.310	2.451	2.498	2.460	2.319	2.401	2.545	2.390	2.483	2.483	2.951	2.953	2.953	3.118	2.964	2.715	2.921	2.907	2.889	3.018	3.176	3.073	2.921
Q9XIF0	1.351	3.285	3.289	3.602	3.540	3.699	2.765	2.977	3.620	3.899	3.643	2.450	2.724	2.778	2.655	2.657	2.676	2.687	2.795	2.716	2.807	2.514	2.550	2.495	2.507	2.518	2.723	2.682	2.642	2.653	2.556	2.990	3.012	2.814	2.921	2.972	2.945	3.064	3.088
Q9STE5	1.529	3.246	3.419	3.471	3.530	3.673	3.168	3.092	3.437	3.558	3.679	2.511	2.777	2.674	2.482	2.464	2.528	2.766	2.491	2.580	2.654	2.622	2.590	2.587	2.634	2.677	3.058	3.026	2.804	2.973	3.026	2.912	2.871	2.840	2.895	3.034	2.928	2.953	3.183
Q42484	1.297	3.382	3.404	3.631	3.526	4.166	3.484	3.467	3.590	3.529	4.356	2.418	2.609	2.350	2.339	2.518	2.310	2.388	2.501	2.621	2.646	2.556	2.461	2.505	2.477	2.552	2.651	2.613	2.726	2.811	2.745	2.797	2.716	2.561	2.789	2.611	3.311	3.491	3.534
Q8RXS5	1.048	3.311	3.412	3.572	3.406	4.001	3.387	3.375	3.578	3.515	3.918	2.357	2.116	2.213	2.215	2.314	2.457	2.202	2.240	2.332	2.298	2.438	2.409	2.514	2.447	2.498	2.867	2.915	2.800	2.769	3.007	2.827	2.823	2.791	2.790	2.798	2.931	2.897	3.102
Q8W474	1.357	3.330	3.349	3.568	3.572	3.770	2.846	3.255	3.580	3.574	3.766	2.498	2.381	2.587	2.635	2.786	2.476	2.404	2.664	2.452	2.651	2.501	2.639	2.592	2.598	2.572	2.916	2.719	2.700	2.818	2.793	2.770	2.822	3.031	2.755	2.863	2.930	3.228	3.118
A0A1P8AP86	1.255	3.346	3.344	3.861	3.678	3.627	3.328	3.341	3.582	3.516	3.562	2.360	2.224	2.566	2.429	2.372	2.163	2.394	2.605	2.270	2.233	2.435	2.511	2.390	2.449	2.491	2.859	2.788	2.777	2.863	2.875	2.711	2.819	2.741	2.914	2.843	3.040	3.053	3.395
Q9C646	1.206	3.197	3.227	3.582	3.842	3.666	2.695	3.006	3.543	3.577	4.035	2.329	2.768	2.458	2.631	2.428	2.550	2.475	2.375	2.545	2.509	2.654	2.626	2.605	2.608	2.632	2.794	2.832	2.738	2.907	2.945	2.834	2.837	2.793	2.741	2.878	3.004	2.983	3.095
A0A5S9WIX4	0.991	3.331	3.424	3.499	3.574	3.695	3.381	3.458	3.656	3.607	3.691	2.324	2.399	2.217	2.219	2.349	2.200	2.366	2.445	2.163	2.260	2.515	2.545	2.573	2.596	2.495	2.865	2.985	2.993	2.956	2.818	2.725	2.955	2.830	2.972	2.892	2.925	3.091	2.998
Q9SX38	1.407	3.287	3.186	3.622	3.632	3.647	3.067	3.176	3.573	4.104	3.676	2.760	2.779	2.871	2.544	2.839	2.808	2.667	2.609	2.684	2.880	2.619	2.536	2.593	2.573	2.653	2.827	2.997	2.788	2.924	3.268	2.876	2.943	2.840	2.987	2.794	2.974	3.236	3.361
Q9LMP6	1.174	3.285	3.353	3.370	3.537	3.941	3.275	3.339	3.820	3.542	3.754	2.184	2.357	2.411	2.279	2.248	2.330	2.321	2.263	2.589	2.427	2.405	2.415	2.477	2.399	2.420	2.678	2.678	2.695	2.606	2.638	2.660	2.669	2.689	2.649	2.779	2.996	3.477	3.025
Q9FG90	1.106	3.327	3.307	3.459	3.576	3.680	3.390	3.341	3.592	3.611	3.683	2.238	2.182	2.176	2.191	2.352	2.270	2.240	2.359	2.278	2.400	2.567	2.581	2.396	2.494	2.506	2.778	3.007	2.791	2.813	2.818	2.830	2.799	2.600	2.943	2.822	2.866	3.075	3.438
P60839	0.989	3.373	3.379	4.469	3.710	3.996	3.514	3.535	3.867	3.636	4.388	2.164	2.113	2.551	2.161	2.179	2.357	2.182	2.192	2.155	2.173	2.457	2.491	2.425	2.425	2.566	2.678	2.790	2.721	2.703	2.720	2.657	2.799	2.608	2.714	2.805	2.961	2.980	2.970
Q39214	1.204	3.003	2.955	3.603	3.595	3.924	2.859	3.138	3.563	3.589	3.895	2.354	2.253	2.356	2.203	2.332	2.372	2.279	2.584	2.277	2.335	2.599	2.604	2.613	2.642	2.599	2.991	2.936	3.012	2.895	2.979	2.850	2.751	2.785	2.618	2.927	3.020	3.018	3.039
Q8W3K3	1.400	3.141	3.011	3.594	3.561	3.743	2.678	2.919	4.356	3.545	3.733	2.298	2.293	2.482	2.324	2.472	2.325	2.569	2.505	2.419	2.637	2.494	2.485	2.591	2.523	2.426	2.863	2.729	2.734	2.689	2.747	2.694	2.699	2.886	2.947	2.771	2.867	3.065	3.001

Table S2 – MolProbity scores after geometry optimization. Note the difference for the RFAA models.

	AF2 DB	AF2 AC					AF2 IC					AF2 AM					AF2 IM					AF3 - No Ligand					AF3 - ADP					AF3 - ATP					RFAA - No Ligand	RFAA - ADP	RFAA - ATP
Sequence	Rank 0	Rank 0	Rank 1	Rank 2	Rank 3	Rank 4	Rank 0	Rank 1	Rank 2	Rank 3	Rank 4	Rank 0	Rank 1	Rank 2	Rank 3	Rank 4	Rank 0	Rank 1	Rank 2	Rank 3	Rank 4	Rank 0	Rank 1	Rank 2	Rank 3	Rank 4	Rank 0	Rank 1	Rank 2	Rank 3	Rank 4	Rank 0	Rank 1	Rank 2	Rank 3	Rank 4	Rank 0	Rank 0	Rank 0
Q9LVT3	0.916	1.012	0.966	0.796	0.955	0.813	1.116	1.016	0.796	0.996	0.909	0.939	0.618	0.683	0.957	0.859	0.933	0.722	0.903	0.767	0.805	0.767	0.710	0.859	0.813	0.810	0.768	0.878	0.936	0.802	0.842	0.891	0.808	0.768	0.971	0.919	3.591	1.999	1.911
A0A654FPA2	0.711	0.845	0.817	0.820	0.898	0.903	0.837	0.872	0.861	0.930	1.122	0.540	0.666	0.593	0.737	0.605	0.693	0.561	0.666	0.581	1.092	0.734	0.709	0.537	0.602	0.682	0.687	0.649	0.707	0.649	0.720	0.790	0.823	0.750	0.687	0.752	0.811	1.336	1.067
Q9M667	0.783	0.717	0.889	0.810	0.881	1.622	0.729	0.781	0.922	0.781	2.110	0.806	0.770	0.605	0.875	0.770	0.697	0.591	0.687	0.715	0.730	0.689	0.713	0.697	0.713	0.613	0.836	0.872	0.883	0.793	0.932	0.685	0.695	0.871	0.818	0.833	0.941	0.968	1.100
A0A654EJC3	0.953	1.069	0.941	0.853	0.919	1.389	0.731	0.731	0.853	0.840	1.518	0.731	0.744	0.794	0.768	0.782	0.836	0.994	0.756	0.717	0.932	0.717	0.665	0.631	0.654	0.679	0.860	0.813	0.860	0.716	0.819	0.815	0.757	0.922	0.800	0.859	0.815	1.021	0.958
A0A654EJG6	0.916	0.909	0.886	0.853	0.829	1.086	0.835	0.788	0.763	2.243	0.930	0.720	0.816	0.679	0.810	0.775	0.728	0.674	0.773	0.738	0.866	0.661	0.578	0.591	0.578	0.620	0.822	0.876	0.733	0.734	0.952	0.812	0.861	0.773	0.775	0.773	0.940	1.034	1.393
Q9FLB4	0.685	1.200	1.003	1.288	0.640	0.951	1.074	1.033	1.819	0.685	0.894	0.679	0.669	0.700	0.741	0.734	0.634	0.593	0.685	0.774	0.741	0.623	0.685	0.696	0.700	0.685	0.860	0.805	0.900	0.754	0.813	0.916	0.874	0.874	0.860	0.944	3.574	1.336	1.334
Q8W3K0	0.840	1.167	1.151	0.863	0.830	1.111	1.478	1.328	0.808	0.863	1.746	0.767	0.705	0.724	0.943	0.899	0.776	0.698	0.850	0.714	0.694	0.627	0.776	0.725	0.697	0.659	0.825	0.711	0.681	0.777	0.776	0.791	0.806	0.624	0.708	0.732	2.490	3.038	2.702
Q38834	0.662	0.853	0.604	0.774	0.857	0.835	0.694	0.612	0.754	0.835	0.919	0.587	0.560	0.675	0.660	0.637	0.636	0.529	0.684	0.700	0.608	0.608	0.608	0.675	0.585	0.655	0.716	0.733	0.655	0.675	0.769	0.716	0.716	0.695	0.723	0.675	0.705	1.208	0.965
Q9LQ54	0.862	0.967	0.892	0.747	0.753	0.795	0.849	0.739	0.781	0.777	0.804	0.796	0.875	0.729	0.803	0.860	0.817	0.868	0.813	0.792	0.768	0.780	0.817	0.858	0.728	0.681	0.751	0.847	0.908	0.774	0.944	0.827	0.862	0.812	0.876	0.828	1.098	3.302	1.232
Q9LRR4	0.807	1.051	1.073	0.978	0.782	1.047	0.988	1.094	0.924	0.849	1.066	0.797	0.890	0.885	0.964	0.859	0.765	0.778	1.342	1.006	0.910	0.828	0.788	0.749	0.822	0.738	0.904	0.875	0.857	0.885	0.917	0.952	0.794	0.787	0.849	0.800	1.539	1.298	1.299
Q9STE7	0.803	1.122	0.966	0.776	2.053	0.976	0.937	0.749	1.512	0.829	1.052	0.659	0.681	0.801	0.827	0.664	0.764	0.812	0.761	0.652	0.628	0.783	0.581	0.581	0.707	0.812	0.879	0.875	0.808	0.770	0.747	0.926	0.944	0.939	0.828	0.950	0.867	3.147	1.073
A0A5S9WPD4	0.801	1.278	1.208	0.791	0.885	1.385	1.209	1.224	1.364	0.978	1.118	0.713	0.807	0.789	0.652	0.747	0.676	0.747	0.774	0.746	0.832	0.672	0.791	0.677	0.746	0.645	0.992	0.912	0.804	0.901	0.883	0.918	0.891	0.873	0.898	0.925	4.578	4.664	3.379
Q8W4J9	0.671	0.956	0.883	0.912	0.959	0.761	0.809	0.685	1.065	0.851	0.877	0.598	0.792	0.625	0.656	0.608	0.529	0.654	0.713	0.569	0.719	0.734	0.656	0.681	0.699	0.603	0.844	0.791	0.648	0.731	0.711	0.828	0.703	0.784	0.809	0.807	0.955	1.105	1.339
O64973	0.822	0.933	0.851	0.797	0.917	0.837	0.957	1.011	0.797	0.877	0.880	0.720	0.598	0.757	0.734	0.721	0.649	0.672	0.692	0.662	0.734	0.679	0.607	0.721	0.746	0.720	0.765	0.851	0.839	0.836	0.763	0.934	0.783	0.855	0.873	0.820	1.257	1.924	1.011
Q9SI85	0.744	0.905	0.950	3.528	0.812	0.981	1.004	1.041	0.949	0.796	0.955	0.732	0.660	0.767	0.738	0.718	0.625	0.691	0.718	0.732	0.703	0.648	0.648	0.690	0.744	0.703	0.818	0.900	0.896	0.863	0.849	0.973	0.853	0.868	0.818	0.818	0.898	1.490	1.356
Q8L3R3	0.862	1.007	1.044	0.881	0.790	0.984	0.907	0.928	0.859	0.894	0.845	0.775	0.759	0.716	2.349	0.681	0.811	0.722	0.815	0.707	0.747	0.680	0.732	0.747	0.664	0.591	0.743	0.776	0.855	0.743	0.769	0.842	0.821	0.789	0.807	0.782	1.035	1.644	1.095
Q940K0	0.649	0.933	0.865	0.873	0.920	0.939	0.925	0.991	0.878	0.934	0.971	0.845	0.748	0.645	0.748	0.705	0.678	0.875	0.585	0.861	0.878	0.731	0.784	0.672	0.654	0.689	0.922	0.887	0.803	0.803	0.834	0.822	0.940	0.874	0.786	0.865	1.765	2.211	1.505
P60838	0.687	0.851	0.851	0.856	1.381	0.804	0.869	0.835	0.864	0.879	0.827	0.691	0.782	0.631	0.554	0.771	0.632	0.691	0.631	0.744	0.804	0.676	0.705	0.746	0.705	0.631	0.777	0.849	0.801	0.877	0.781	0.889	0.777	0.889	0.829	0.814	0.928	1.036	1.121
O82484	0.661	0.767	0.892	0.749	0.804	0.787	0.778	0.825	0.577	1.101	0.703	0.644	0.706	0.652	0.644	0.677	0.555	0.625	0.577	0.584	0.584	0.532	0.606	0.577	0.584	0.560	0.667	0.802	0.687	0.761	0.699	0.705	0.820	0.746	0.763	0.649	2.012	1.872	1.494
Q9XIF0	0.682	1.137	0.988	0.894	0.773	0.940	0.893	0.754	0.859	1.029	0.874	0.671	0.500	0.919	0.888	0.985	0.799	0.744	0.977	0.909	2.903	0.686	0.602	0.763	0.667	0.649	0.957	0.799	0.971	0.895	0.844	0.754	0.863	0.811	0.703	0.895	1.358	1.111	1.107
Q9STE5	0.745	1.003	0.852	0.722	0.795	0.844	0.785	0.710	0.722	0.805	0.837	0.822	0.529	0.812	0.776	0.652	0.708	0.697	0.774	0.738	0.724	0.738	0.683	0.725	0.555	0.790	0.751	0.683	0.770	0.771	0.730	0.791	0.902	0.846	0.747	0.827	0.875	1.065	1.231
Q42484	0.696	0.846	1.055	0.893	0.837	1.082	0.809	1.020	0.709	0.781	2.000	0.709	0.656	0.697	0.684	0.684	0.684	0.697	0.740	0.808	0.742	0.699	0.624	0.685	0.602	0.589	0.771	0.885	0.813	0.768	0.664	0.813	0.768	0.810	0.753	0.820	2.428	2.664	2.407
Q8RXS5	0.761	0.844	0.901	0.843	0.880	1.046	0.976	0.837	0.826	0.780	0.972	0.785	0.665	0.796	0.708	0.799	0.780	0.746	0.740	0.779	2.392	0.650	0.663	0.761	0.607	0.734	0.929	0.879	0.923	0.822	0.846	0.851	0.859	0.859	0.917	0.947	1.217	1.197	2.138
Q8W474	0.777	1.009	0.815	0.895	0.815	0.911	0.697	0.765	0.845	0.852	0.984	0.654	0.799	0.686	0.803	0.703	0.727	0.657	0.625	0.844	0.822	0.618	0.686	0.686	0.529	0.810	0.916	0.916	0.841	0.861	0.889	0.835	0.889	0.935	0.772	0.860	0.988	1.099	1.033
A0A1P8AP86	0.822	0.933	0.962	0.837	0.982	0.822	0.951	0.844	0.822	0.865	0.858	0.721	0.634	0.786	2.522	0.804	0.773	0.832	0.705	0.849	0.563	0.834	0.821	0.721	0.680	0.731	0.888	0.945	0.836	0.763	0.866	0.916	1.002	0.945	0.898	0.840	1.489	1.520	2.157
Q9C646	0.689	0.775	0.951	0.818	0.958	0.902	0.703	0.753	0.775	0.915	4.077	0.685	0.720	0.578	0.791	0.742	0.675	0.580	0.752	0.804	0.738	0.671	0.578	0.651	0.651	0.602	0.737	0.674	0.698	0.666	0.878	0.793	0.754	0.736	0.648	0.692	0.858	1.053	3.171
A0A5S9WIX4	0.713	0.990	0.900	0.802	0.882	0.842	0.834	0.900	0.811	0.802	0.923	0.734	0.656	0.670	0.500	0.668	0.684	0.722	0.684	0.640	0.668	0.698	0.713	0.711	0.605	0.652	0.849	0.772	0.900	0.708	0.928	0.692	0.690	0.749	0.854	0.845	0.908	1.975	1.463
Q9SX38	0.860	1.089	0.829	0.851	0.791	0.894	0.902	0.772	0.772	1.464	0.801	0.809	0.741	0.940	0.908	0.889	0.759	0.829	0.770	0.832	0.918	0.707	0.692	0.734	0.812	0.691	0.898	1.019	0.945	0.877	0.996	0.959	0.839	0.983	0.826	0.936	1.508	1.224	1.569
Q9LMP6	0.695	1.037	0.937	0.681	0.804	1.014	1.015	0.935	1.376	0.835	0.884	0.710	0.695	0.804	0.751	0.800	0.779	0.663	0.701	0.679	0.785	0.666	0.792	0.676	0.679	0.613	0.832	0.791	1.007	0.985	0.870	0.856	0.887	0.936	0.892	0.977	0.783	2.026	1.158
Q9FG90	0.732	0.823	0.827	0.771	0.807	0.916	0.879	0.780	0.867	0.853	0.904	0.783	0.779	0.676	0.690	0.746	0.826	0.690	0.705	0.750	0.745	0.629	0.690	0.640	0.611	0.640	0.834	0.781	0.887	0.872	0.976	0.818	0.908	0.887	0.872	0.876	1.031	1.886	2.556
P60839	0.647	0.902	0.953	2.477	0.815	1.092	1.000	0.908	0.958	0.891	1.760	0.539	0.536	0.536	0.500	0.500	0.529	2.266	0.500	0.500	0.529	0.539	0.565	0.636	0.500	0.600	0.730	0.770	0.789	0.729	0.641	0.892	0.808	0.907	0.670	0.770	1.045	0.997	1.822
Q39214	0.652	0.715	0.845	0.867	0.916	1.026	0.905	0.891	0.845	0.861	1.059	0.628	0.755	0.679	0.682	0.747	0.747	0.715	0.591	2.370	0.658	0.591	0.598	0.552	0.702	0.578	0.721	0.796	0.729	0.814	0.735	0.716	0.733	0.747	0.733	0.681	1.303	0.989	1.019
Q8W3K3	0.885	0.882	0.921	0.742	0.894	0.940	0.820	0.786	2.009	0.798	0.876	0.710	0.597	0.893	0.731	0.864	0.578	0.684	0.840	0.666	0.926	0.666	0.732	0.702	0.644	0.624	0.857	0.710	0.821	0.727	0.769	0.769	0.874	0.883	0.806	0.664	0.809	1.096	1.055

Table S3 – Maximum RMSD for ZAR1 models, during a 10ns MD simulation

Model Name	Maximum RMSD (Å)
Active Crystal	3.317
Inactive Crystal	3.768
AF2 – Database	3.223
AF2 – Active Control	3.649
AF2 – Inactive Control	2.926
AF2 – Active MSA	3.873
AF2 – Inactive MSA	2.879
AF3 – ADP	2.837
AF3 – ATP	3.145
AF3 – No Ligand	2.454
RFAA – ADP	3.318
RFAA – ATP	2.692
RFAA – No Ligand	2.921

Table S4 – Maximum RMSD (Å), for the wider CNL protein set, obtained by enhanced sampling using Robosample.

Sequence	AF2 Database	AF2 Active MSA	AF2 Inactive MSA	AF3
A0A5S9WIX4	2.822	2.365	1.910	1.872
A0A654EJC3	2.644	1.717	2.403	2.071
A0A654EJG6	0.644	2.042	2.597	2.373
A0A654FPA2	2.297	2.708	2.026	1.824
O64973	2.153	2.253	2.868	2.537
O82484	0.688	1.944	2.524	0.266
P60838	2.459	0.616	2.121	2.436
P60839	2.305	1.928	2.355	2.132
Q38834	0.674	1.770	2.438	0.332
Q39214	0.593	0.605	1.980	0.290
Q42484	2.209	2.356	2.189	1.917
Q8L3R3	2.007	2.804	0.615	2.742
Q8RXS5	2.543	2.235	2.414	2.439
Q8W3K0	0.661	2.350	2.553	1.715
Q8W3K3	1.941	0.625	2.184	1.962
Q8W474	2.292	1.824	2.390	2.468
Q8W4J9	1.814	2.243	2.205	2.047
Q940K0	2.206	2.139	1.952	2.146
Q9C646	1.748	1.611	0.614	1.668
Q9FG90	2.289	0.634	2.496	2.179
Q9FLB4	2.010	1.768	2.017	2.435
Q9LMP6	2.785	2.084	2.701	2.453
Q9LQ54	2.364	2.171	2.841	0.314
Q9LRR4	0.636	2.604	2.189	2.685
Q9LVT3	0.592	1.780	1.871	0.327
Q9M667	2.169	3.425	2.895	2.506
Q9SI85	2.061	2.387	1.987	2.101
Q9STE5	2.512	2.283	0.619	1.688
Q9STE7	2.010	2.371	2.819	0.320
Q9SX38	2.184	2.619	2.146	0.331
Q9XIF0	3.392	2.488	1.781	0.375

Table S5 – Coordinates for CC, ARC2 and LRR centers of mass, as well as for the Origin Plane, corresponding to Figure 7 of the main text. All positions are given in nanometers.

		VG			NBD Center			ARC1 Center		
Origin Plane		0	0	0	-2.38	0	0	0.643	1.818	0

	**Alphafold 3 - ADP**									
EDVID	Q38834:	-0.547	4.62	-1.009	-0.945	2.478	-0.846	-2.79	3.435	-1.775
	Q8W4J9:	-0.997	4.853	-0.673	-1.155	2.507	-0.589	-3.27	3.573	-0.889
	Q8W3K3:	-2.453	3.242	2.686	-1.061	1.513	2.364	-0.179	2.26	4.704
	Q9LQ54:	-0.914	4.764	-0.314	-1.057	2.62	-0.668	-3.393	3.491	-1.071
	A0A654EJC3:	-0.771	4.827	-0.834	-1.019	2.566	-0.807	-3.262	3.532	-1.772
	Q9SX38:	-2.358	2.816	3.11	-1.079	1.191	2.484	-0.084	1.552	5.202
	Q8W474:	-0.666	4.933	-0.884	-1.082	2.657	-0.771	-3.212	3.737	-1.191
	Q9C646:	-2.295	3.728	2.984	-1.09	1.566	2.308	0.012	2.757	4.152
	Q8W3K0:	-1.232	4.746	-0.589	-1.227	2.608	-0.712	-3.497	3.473	-1.115
	Q9XIF0:	-2.462	2.844	2.868	-1.054	1.177	2.523	0.048	2.015	4.568
	Q9STE7:	-1.008	4.583	-0.979	-1.038	2.49	-0.862	-3.27	3.078	-1.896
	A0A654EJG6:	-0.879	4.783	-1.265	-1.111	2.524	-0.927	-3.293	3.343	-1.603
	Q9M667:	-0.845	4.604	-0.718	-1.088	2.497	-0.761	-3.244	3.269	-1.527
	Q39214:	-1.884	3.31	2.638	-0.798	1.444	2.344	0.514	1.814	4.45
	Q9LRR4:	-1.899	3.304	2.918	-1.012	1.611	2.243	0.321	2.082	4.107
	Q9STE5:	-0.479	4.477	-1.574	-0.85	2.343	-1.185	-2.93	3.018	-2.544
RPS5	O64973:	-3.445	-2.146	-4.411	-1.002	2.62	-0.774	-3.126	3.785	-0.92
	Q940K0:	-2.812	-4.278	-2.046	-0.96	1.654	2.17	0.302	2.648	3.953
	Q8L3R3:	-5.324	1.034	-2.718	-1.045	2.713	-0.554	-3.248	3.734	-0.585
	Q42484:	-6.064	0.544	-2.203	-0.821	1.652	2.172	0.573	2.632	3.948
	Q9FG90:	-5.73	0.683	-1.962	-1.069	2.685	-0.677	-3.182	3.84	-0.768
	P60839:	-2.438	-3.033	1.114	-1.126	2.597	-0.691	-3.252	3.674	-0.716
	Q9LMP6:	-5.415	0.329	-4.03	-1.151	2.67	-0.69	-3.394	3.629	-0.765
	Q9SI85:	-4.935	1.281	-3.498	-1.166	2.543	-0.927	-3.244	3.393	-1.342
	A0A654FPA2:	-5.173	1.412	-2.848	-1.111	2.641	-0.606	-3.252	3.721	-0.628
	Q8RXS5:	-4.741	1.88	-2.946	-1.153	2.568	-0.833	-3.308	3.596	-1.281
	Q9FLB4:	-5.762	1.041	-2.957	-1.08	2.575	-0.61	-3.235	3.661	-0.876
	O82484:	-4.497	1.521	-2.777	-1.139	2.633	-0.765	-3.313	3.61	-1.012
	P60838:	-5.74	0.78	-3.242	-1.039	2.7	-0.588	-3.195	3.813	-0.595
	A0A1P8AP86:	-5.21	-0.353	-3.965	-0.809	1.564	2.24	0.496	2.674	3.919
	A0A5S9WIX4:	-5.207	0.687	-4.019	-1.059	2.697	-0.64	-3.24	3.697	-0.774
	Q9LVT3:	2.158	2.789	-3.303	-0.857	2.657	-0.719	-3.081	3.394	-0.851
Non A. Thaliana	Q6WSR5:	-1.772	4.036	2.41	-1.054	1.932	1.988	0.312	3.175	3.335
	Q6WWJ4:	-2.135	4.628	-1.923	-1.559	2.723	-1.237	-3.724	2.713	-2.173
	Q9S7Q4:	-0.778	4.387	-1.355	-1.087	2.455	-0.953	-3.554	3.073	-2.306
	Q15J16:	-1.011	4.507	-1.169	-1.103	2.447	-0.985	-3.107	3.346	-1.759
	**Alphafold 3 - ATP**									
EDVID	Q38834:	-0.516	4.661	-0.973	-0.939	2.515	-0.821	-2.801	3.5	-1.682
	Q8W4J9:	-2.284	3.178	2.867	-0.95	1.42	2.317	0.017	2.337	4.314
	Q8W3K3:	-0.633	4.866	-0.657	-1.04	2.649	-0.652	-3.18	3.745	-1.6
	Q9LQ54:	-1.09	4.736	-0.28	-1.118	2.64	-0.656	-3.451	3.465	-1.12
	A0A654EJC3:	-0.645	4.77	-1.019	-0.952	2.529	-0.93	-3.152	3.505	-1.99
	Q9SX38:	-2.26	2.944	3.009	-1.069	1.176	2.467	0.004	1.384	5.2
	Q8W474:	-0.776	4.793	-0.749	-1.053	2.626	-0.703	-3.18	3.672	-1.075
	Q9C646:	-0.815	4.71	-1.038	-1.049	2.516	-0.913	-3.222	3.478	-1.55
	Q8W3K0:	-1.122	4.672	-0.684	-1.139	2.595	-0.853	-3.32	3.514	-1.481
	Q9XIF0:	-0.403	4.645	-0.754	-1.024	2.559	-0.685	-3.173	3.641	-1.012
	Q9STE7:	-0.996	4.636	-0.849	-1.017	2.519	-0.848	-3.248	3.109	-1.864
	A0A654EJG6:	-0.871	4.808	-1.116	-1.055	2.534	-0.92	-3.216	3.398	-1.637
	Q9M667:	-0.759	4.574	-0.899	-1.054	2.479	-0.822	-3.178	3.241	-1.69
	Q39214:	-1.726	3.619	3.12	-0.79	1.443	2.326	0.576	1.834	4.375
	Q9LRR4:	-1.875	3.272	3.026	-0.993	1.587	2.265	0.352	2.106	4.146
	Q9STE5:	-0.474	4.559	-1.526	-0.878	2.442	-1.114	-2.967	3.229	-2.405
RPS5	O64973:	-3.675	-2.003	-4.169	-1.016	2.653	-0.722	-3.115	3.886	-0.763
	Q940K0:	-4.795	0.997	-3.462	-1.141	2.628	-0.88	-3.33	3.532	-1.378
	Q8L3R3:	-5.3	1.399	-2.872	-1.034	2.682	-0.548	-3.233	3.691	-0.602
	Q42484:	-3.718	-1.098	-4.309	-0.825	1.642	2.181	0.51	2.752	3.924
	Q9FG90:	-5.947	0.047	-1.78	-1.074	2.649	-0.705	-3.189	3.789	-0.901
	P60839:	-3.204	-2.872	0.92	-1.136	2.613	-0.682	-3.296	3.644	-0.614
	Q9LMP6:	-6.167	0.556	-2.7	-1.056	2.666	-0.736	-3.271	3.698	-0.895
	Q9SI85:	-4.553	0.927	-3.798	-1.131	2.593	-0.869	-3.211	3.469	-1.224
	A0A654FPA2:	-4.733	1.122	-3.211	-1.046	2.643	-0.668	-3.14	3.773	-0.8
	Q8RXS5:	-5.125	-1.48	-3.904	-0.955	1.863	2.112	0.366	3.025	3.814
	Q9FLB4:	-4.525	0.371	-4.913	-0.934	2.609	-0.687	-3.052	3.745	-1.049
	O82484:	-4.523	1.925	-2.725	-1.099	2.674	-0.713	-3.248	3.731	-0.95
	P60838:	-5.86	0.631	-3.29	-0.995	2.662	-0.667	-3.192	3.683	-0.775
	A0A1P8AP86:	-4.979	-1.904	-3.676	-1.075	2.602	-0.739	-3.2	3.741	-0.972
	A0A5S9WIX4:	-5.134	0.18	-4.044	-1.114	2.689	-0.666	-3.295	3.688	-0.816
	Q9LVT3:	0.876	4.015	-3.995	-0.899	2.733	-0.55	-3.119	3.493	-0.494
Non A. Thaliana	Q6WSR5:	-1.852	4.068	2.357	-1.058	1.974	1.986	0.26	3.279	3.308
	Q6WWJ4:	-2.197	4.674	-1.733	-1.595	2.752	-1.211	-3.797	2.753	-2.066
	Q9S7Q4:	-0.736	4.363	-1.49	-1.078	2.446	-0.991	-3.523	3.122	-2.403
	Q15J16:	-1.009	4.547	-1.039	-1.097	2.486	-0.934	-3.089	3.421	-1.682

Alphafold 3 - No Ligand

EDVID	Q38834:	-0.428	4.655	-0.854	-0.87	2.499	-0.756	-2.75	3.512	-1.567
	Q8W4J9:	-0.674	4.852	-0.626	-1.033	2.56	-0.564	-3.103	3.741	-0.912
	Q8W3K3:	-2.276	3.163	2.795	-0.987	1.365	2.409	-0.03	1.875	4.767
	Q9LQ54:	-0.53	4.806	0.011	-0.969	2.722	-0.607	-3.154	3.855	-0.976
	A0A654EJC3:	-2.183	3.224	2.785	-0.999	1.333	2.395	0.049	1.996	4.73
	Q9SX38:	-0.758	4.809	0.248	-1.058	2.687	-0.169	-3.528	4.148	-0.65
	Q8W474:	-0.533	4.838	-0.657	-0.91	2.633	-0.751	-3.012	3.803	-1.126
	Q9C646:	-2.226	3.32	2.706	-1.016	1.397	2.412	0.089	2.43	4.343
	Q8W3K0:	-0.969	4.766	-0.611	-1.197	2.582	-0.682	-3.426	3.572	-1.034
	Q9XIF0:	-2.541	2.707	2.865	-1.063	1.094	2.558	-0.062	1.916	4.694
	Q9STE7:	-0.695	4.657	-0.468	-0.895	2.543	-0.699	-3.096	3.414	-1.701
	A0A654EJG6:	-2.514	3.168	2.769	-1.157	1.371	2.416	-0.154	2.301	4.43
	Q9M667:	-0.674	4.658	-0.584	-0.989	2.55	-0.698	-3.097	3.457	-1.504
	Q39214:	-1.839	3.206	2.656	-0.699	1.411	2.367	0.688	1.765	4.466
	Q9LRR4:	-1.967	3.2	3.116	-0.989	1.529	2.281	0.267	2.154	4.221
	Q9STE5:	-0.27	4.532	-1.114	-0.733	2.401	-0.937	-2.889	3.425	-2
RPS5	O64973:	-5.685	0.286	-3.102	-0.957	1.606	2.208	0.322	2.737	3.922
	Q940K0:	-4.849	-2.427	-4.058	-0.966	1.624	2.191	0.282	2.624	3.987
	Q8L3R3:	-5.613	-1.005	-3.175	-0.961	1.607	2.242	0.361	2.676	3.986
	Q42484:	-2.822	-2.134	-4.082	-0.82	1.591	2.207	0.567	2.605	3.973
	Q9FG90:	-2.754	-2.276	-4.676	-0.94	2.78	-0.624	-2.97	4.096	-0.664
	P60839:	-2.097	-3.691	0.56	-1.046	2.694	-0.684	-3.098	3.912	-0.585
	Q9LMP6:	-2.929	-0.233	-5.426	-0.998	1.82	2.17	0.253	3.039	3.873
	Q9SI85:	-5.418	0.997	-3.613	-0.922	1.622	2.223	0.35	2.625	3.82
	A0A654FPA2:	-2.625	-0.926	-5.134	-0.852	1.585	2.224	0.49	2.582	3.952
	Q8RXS5:	-5.315	-1.316	-3.647	-1.065	1.919	2.058	0.182	3.149	3.767
	Q9FLB4:	-2.291	-3.186	-4.47	-0.975	2.696	-0.604	-3.039	3.985	-0.758
	O82484:	-4.287	-2.018	-4.117	-1.047	1.649	2.237	0.292	2.696	3.948
	P60838:	-5.453	-0.035	-3.883	-0.873	2.718	-0.618	-3.016	3.865	-0.716
	A0A1P8AP86:	-5.28	-1.617	-3.977	-0.859	1.616	2.23	0.518	2.696	3.89
	A0A5S9WIX4:	-2.917	-2.477	-4.441	-0.905	1.518	2.241	0.472	2.477	3.989
	Q9LVT3:	-0.89	4.878	-4.657	-0.924	2.792	-0.475	-3.098	3.659	-0.359
Non A. Thaliana	Q6WSR5:	-1.797	3.841	2.453	-0.92	1.807	2.086	0.415	3.142	3.396
	Q6WWJ4:	-1.843	4.728	-1.643	-1.425	2.748	-1.131	-3.658	2.958	-1.889
	Q9S7Q4:	-0.716	4.638	-0.783	-1.132	2.611	-0.71	-3.626	3.579	-1.696
	Q15J16:	-1.724	3.554	2.578	-0.981	1.7	2.303	0.04	2.633	4.158

AF2 - Database

EDVID	Q38834:	-0.462	4.644	-1.015	-0.91	2.487	-0.827	-2.791	3.45	-1.72
	Q8W4J9:	-0.926	4.694	-0.326	-0.923	2.555	-0.737	-2.988	3.772	-1.264
	Q8W3K3:	-0.59	4.823	-0.337	-0.878	2.664	-0.722	-2.901	3.994	-1.72
	Q9LQ54:	-0.993	4.615	0.009	-0.908	2.678	-0.721	-3.093	3.788	-1.26
	A0A654EJC3:	-0.605	4.801	-0.257	-0.758	2.657	-0.897	-2.79	3.93	-2.045
	Q9SX38:	-0.81	4.786	0.084	-0.829	2.698	-0.545	-3.088	4.115	-1.622
	Q8W474:	-0.575	4.814	-0.515	-1.006	2.615	-0.713	-3.076	3.847	-0.999
	Q9C646:	-0.611	4.748	-0.407	-1.034	2.563	-0.684	-3.135	3.783	-1.014
	Q8W3K0:	-1.006	4.765	-0.513	-1.19	2.525	-0.712	-3.369	3.556	-1.208
	Q9XIF0:	-0.442	4.634	-0.684	-1.031	2.542	-0.751	-3.212	3.62	-1.206
	Q9STE7:	-0.824	4.578	-0.684	-0.921	2.457	-0.802	-3.131	3.17	-1.917
	A0A654EJG6:	-0.809	4.86	-0.264	-0.859	2.719	-0.864	-2.915	3.909	-1.608
	Q9M667:	-0.631	4.701	-0.615	-0.929	2.549	-0.718	-2.996	3.492	-1.591
	Q39214:	-1.803	3.455	2.417	-0.787	1.549	2.272	0.466	1.907	4.45
	Q9LRR4:	-1.831	3.133	3.059	-1.017	1.46	2.301	0.294	2.002	4.242
	Q9STE5:	-0.256	4.506	-1.327	-0.722	2.418	-1.059	-2.828	3.335	-2.292
RPS5	O64973:	-5.661	-0.094	-3.458	-0.873	1.546	2.234	0.479	2.57	3.982
	Q940K0:	-3.171	-0.419	-5.457	-0.943	1.555	2.216	0.358	2.462	4.026
	Q8L3R3:	-4.784	0.275	-4.519	-0.915	2.741	-0.668	-3.024	3.904	-0.959
	Q42484:	-2.991	-0.679	-4.979	-0.752	1.506	2.223	0.722	2.374	4.009
	Q9FG90:	-2.65	-2.855	-4.272	-0.936	2.75	-0.72	-3.071	3.937	-0.835
	P60839:	-1.761	-3.321	-3.954	-1.062	2.713	-0.53	-3.141	3.865	-0.198
	Q9LMP6:	-2.69	-0.568	-5.437	-0.918	1.681	2.198	0.387	2.749	3.961
	Q9SI85:	-3.109	-1.015	-5.092	-0.843	1.528	2.238	0.523	2.474	3.83
	A0A654FPA2:	-5.197	0.342	-3.904	-0.931	2.757	-0.605	-3.028	3.924	-0.722
	Q8RXS5:	-3.147	-0.95	-5.313	-1.023	1.855	2.041	0.267	2.965	3.825
	Q9FLB4:	-3.013	-0.681	-5.381	-0.866	1.717	2.118	0.384	2.87	3.856
	O82484:	-5.364	-0.109	-3.402	-1.003	1.654	2.209	0.365	2.69	3.914
	P60838:	-2.962	0.168	-5.312	-0.784	1.543	2.221	0.677	2.497	3.922
	A0A1P8AP86:	-5.47	-0.287	-3.896	-0.832	1.476	2.276	0.517	2.466	4.007
	A0A5S9WIX4:	-5.265	-0.284	-3.851	-0.966	2.783	-0.625	-3.077	3.94	-0.733
	Q9LVT3:	-5.208	-1.868	-3.316	-0.673	2.731	-0.882	-2.759	3.814	-1.21
Non A. Thaliana	Q6WSR5:	-0.748	4.78	-1.121	-0.886	2.48	-1.024	-2.954	3.487	-1.299
	Q6WWJ4:	-1.916	4.94	-1.649	-1.476	2.795	-1.169	-3.692	2.966	-1.961
	Q9S7Q4:	-0.675	4.611	-0.74	-1.14	2.662	-0.746	-3.602	3.869	-1.831
	Q15J16:	Not Present in Alphafold Database								

Alphafold 2 - Active MSA

EDVID	Q38834:	-2.435	4.01	2.871	-1.208	1.545	2.273	-0.374	2.293	4.251
	Q8W4J9:	-2.272	3.185	2.855	-0.995	1.411	2.311	0.01	2.307	4.285
	Q8W3K3:	-2.2	3.199	2.912	-0.976	1.3	2.401	-0.009	1.782	4.767
	Q9LQ54:	-0.852	4.64	0.118	-0.861	2.742	-0.706	-2.984	3.989	-1.154
	A0A654EJC3:	-2.117	3.417	2.524	-1.032	1.466	2.326	0.002	2.24	4.619
	Q9SX38:	-0.826	4.803	0.029	-0.788	2.699	-0.552	-2.68	3.931	-2.122
	Q8W474:	-2.3	3.307	2.639	-1.046	1.467	2.356	0.051	2.547	4.232
	Q9C646:	-2.144	3.432	2.599	-1.059	1.435	2.337	0.033	2.417	4.291
	Q8W3K0:	-2.322	3.447	2.641	-1.093	1.584	2.34	-0.055	2.624	4.301
	Q9XIF0:	-2.351	2.997	2.632	-1.08	1.193	2.496	0.006	2.021	4.554
	Q9STE7:	-0.866	4.746	-0.639	-0.869	2.494	-0.766	-3.042	3.319	-1.912
	A0A654EJG6:	-2.4	3.319	2.746	-1.116	1.466	2.367	-0.055	2.495	4.344
	Q9M667:	-0.654	4.567	-0.613	-0.959	2.491	-0.781	-3.022	3.392	-1.711
	Q39214:	-1.821	3.511	2.354	-0.824	1.595	2.258	0.377	1.99	4.475
	Q9LRR4:	-1.845	3.246	2.956	-1.052	1.552	2.263	0.211	2.13	4.225
	Q9STE5:	-0.149	4.458	-1.408	-0.68	2.403	-1.104	-2.735	3.357	-2.401
RPS5	O64973:	-3.319	-0.239	-5.209	-0.911	1.485	2.261	0.377	2.487	4.059
	Q940K0:	-3.31	-0.535	-5.398	-0.949	1.628	2.179	0.334	2.577	3.988
	Q8L3R3:	-3.03	-0.189	-5.22	-0.923	1.548	2.248	0.416	2.489	4.04
	Q42484:	-5.676	-0.802	-3.451	-0.681	1.464	2.263	0.775	2.352	4.034
	Q9FG90:	-2.828	-0.922	-5.169	-0.945	1.714	2.18	0.415	2.833	3.846
	P60839:	-1.656	-3.429	-3.855	-1.003	2.696	-0.595	-3.065	3.882	-0.365
	Q9LMP6:	-2.59	-0.67	-5.478	-1.036	1.795	2.161	0.245	2.94	3.899
	Q9SI85:	-3.237	-0.945	-5.097	-0.884	1.59	2.217	0.446	2.592	3.806
	A0A654FPA2:	-3.184	-0.558	-4.982	-0.878	1.524	2.243	0.459	2.475	4.01
	Q8RXS5:	-3.327	-1.57	-5.076	-1.035	1.869	2.042	0.232	2.994	3.827
	Q9FLB4:	-3.935	-1.327	-4.996	-0.784	2.596	-0.874	-2.862	3.744	-1.511
	O82484:	-2.454	-1.44	-5.204	-1.108	1.695	2.233	0.209	2.778	3.943
	P60838:	-3.086	0.221	-5.298	-0.781	1.558	2.213	0.651	2.526	3.927
	A0A1P8AP86:	-3.396	-0.896	-5.077	-0.823	1.502	2.249	0.437	2.567	4.014
	A0A5S9WIX4:	-2.881	-1.587	-4.679	-0.977	1.645	2.194	0.39	2.662	3.909
	Q9LVT3:	-4.41	-2.37	-3.596	-0.677	2.811	-0.75	-2.743	3.925	-0.98
Non A. Thaliana	Q6WSR5:	-2.101	3.934	2.161	-1.064	1.96	1.974	0.116	3.427	3.306
	Q6WWJ4:	-2.708	3.766	2.487	-1.31	2.155	2.058	-0.398	3.534	3.72
	Q9S7Q4:	-0.683	4.619	-0.606	-1.102	2.69	-0.688	-3.542	3.941	-1.793
	Q15J16:	-0.98	4.558	-0.876	-1.072	2.504	-0.896	-3.082	3.495	-1.512

Alphafold 2 - Inactive MSA

EDVID	Q38834:	-0.556	4.715	-1.018	-0.956	2.52	-0.858	-2.828	3.511	-1.703
	Q8W4J9:	-0.738	4.794	-0.707	-1.051	2.515	-0.697	-3.14	3.643	-1.181
	Q8W3K3:	-0.596	4.88	-0.639	-0.991	2.581	-0.717	-3.14	3.738	-1.642
	Q9LQ54:	-0.73	4.8	-0.258	-1.01	2.621	-0.703	-3.24	3.617	-1.218
	A0A654EJC3:	-0.508	4.803	-0.617	-0.907	2.596	-0.801	-3.019	3.779	-1.881
	Q9SX38:	-0.711	4.816	-0.237	-0.986	2.65	-0.462	-3.302	3.987	-1.519
	Q8W474:	-0.581	4.809	-0.596	-0.989	2.633	-0.745	-3.058	3.852	-1.148
	Q9C646:	-0.506	4.82	-0.467	-0.945	2.624	-0.696	-3.004	3.887	-1.144
	Q8W3K0:	-0.895	4.727	-0.658	-1.129	2.547	-0.773	-3.303	3.583	-1.309
	Q9XIF0:	-0.384	4.603	-0.664	-1.029	2.557	-0.736	-3.183	3.714	-1.141
	Q9STE7:	-0.778	4.743	-0.485	-0.86	2.575	-0.734	-3.02	3.505	-1.816
	A0A654EJG6:	-0.755	4.897	-0.853	-1.091	2.577	-0.819	-3.259	3.604	-1.408
	Q9M667:	-0.568	4.662	-0.474	-0.944	2.579	-0.714	-2.97	3.576	-1.616
	Q39214:	-1.836	3.308	2.547	-0.771	1.416	2.341	0.44	1.721	4.575
	Q9LRR4:	-1.875	3.181	3.007	-1.026	1.5	2.286	0.269	2.055	4.238
	Q9STE5:	-0.384	4.595	-1.093	-0.761	2.47	-0.986	-2.899	3.408	-2.174
RPS5	O64973:	-2.838	-1.098	-5.135	-0.898	1.556	2.233	0.459	2.554	3.978
	Q940K0:	-2.949	-0.739	-5.484	-0.952	1.583	2.187	0.263	2.552	4.028
	Q8L3R3:	-4.977	-0.088	-4.399	-0.95	2.717	-0.681	-3.071	3.854	-0.989
	Q42484:	-2.89	-0.646	-5.002	-0.761	1.575	2.194	0.631	2.505	3.988
	Q9FG90:	-2.611	-0.871	-5.177	-0.959	1.693	2.18	0.392	2.853	3.82
	P60839:	-2.443	-3.421	-3.872	-0.909	2.7	-0.779	-2.955	3.948	-0.836
	Q9LMP6:	-2.754	-0.581	-5.528	-1.007	1.768	2.157	0.257	2.973	3.868
	Q9SI85:	-3.239	-0.77	-5.108	-0.89	1.583	2.219	0.423	2.588	3.826
	A0A654FPA2:	-3.128	-0.237	-5.079	-0.89	1.525	2.236	0.422	2.52	3.993
	Q8RXS5:	-3.109	-1.211	-4.897	-1.035	1.887	2.02	0.233	3.076	3.768
	Q9FLB4:	-4.103	-1.332	-4.99	-0.823	2.558	-0.897	-2.917	3.674	-1.557
	O82484:	-2.598	-1.306	-5.311	-1.082	1.661	2.251	0.247	2.717	3.971
	P60838:	-2.914	0.402	-5.32	-0.802	1.543	2.224	0.661	2.496	3.918
	A0A1P8AP86:	-3.434	-0.973	-5.105	-0.836	1.515	2.244	0.421	2.578	4.011
	A0A5S9WIX4:	-3.692	-0.254	-5.125	-0.987	1.638	2.189	0.325	2.694	3.929
	Q9LVT3:	-4.754	-2.186	-3.454	-0.671	2.742	-0.906	-2.745	3.826	-1.287
Non A. Thaliana	Q6WSR5:	-2.012	3.935	2.24	-0.98	1.913	1.983	0.251	3.337	3.308
	Q6WWJ4:	-2.001	4.835	-1.817	-1.438	2.783	-1.245	-3.636	2.915	-2.085
	Q9S7Q4:	-0.756	4.574	-0.857	-1.129	2.609	-0.789	-3.573	3.577	-2.018
	Q15J16:	-1.016	4.463	-1.18	-1.066	2.417	-1.04	-3.054	3.329	-1.83

	**Alphafold2 - Active Control**									
EDVID	Q38834:	-2.458	4.095	2.774	-1.238	1.579	2.26	-0.409	2.347	4.235
	Q8W4J9:	-2.293	3.536	3.312	-1.04	1.404	2.286	0.007	2.255	4.259
	Q8W3K3:	-2.391	3.88	2.931	-1.109	1.569	2.302	-0.17	2.298	4.547
	Q9LQ54:	-2.304	4.036	3.14	-1.182	1.596	2.277	-0.164	2.675	4.179
	A0A654EJC3:	-2.179	3.951	2.725	-1.029	1.564	2.284	0.079	2.323	4.493
	Q9SX38:	-1.896	3.49	3.405	-1.074	1.187	2.452	-0.129	1.148	5.118
	Q8W474:	-2.38	3.967	2.632	-1.164	1.679	2.257	-0.104	2.927	4.03
	Q9C646:	-2.249	3.865	2.752	-1.147	1.607	2.286	-0.079	2.785	4.136
	Q8W3K0:	-2.452	4.028	3.001	-1.136	1.677	2.31	-0.085	2.949	4.063
	Q9XIF0:	-2.475	3.657	3.051	-1.146	1.328	2.451	-0.107	2.255	4.359
	Q9STE7:	-1.958	3.794	3.164	-0.928	1.436	2.312	0.985	2.255	4.279
	A0A654EJG6:	-2.331	3.856	3.138	-1.169	1.587	2.304	-0.058	2.663	4.144
	Q9M667:	-1.805	3.465	3.374	-0.878	1.351	2.407	0.188	1.762	4.564
	Q39214:	-1.886	3.851	2.655	-0.921	1.565	2.25	0.223	1.986	4.493
	Q9LRR4:	-2.118	3.691	2.604	-1.079	1.7	2.153	0.163	2.443	4.018
	Q9STE5:	-1.891	3.685	3.137	-0.935	1.504	2.257	0.202	1.995	4.509
RPS5	O64973:	-1.859	4.197	2.821	-0.98	1.512	2.265	0.319	2.211	4.156
	Q940K0:	-1.397	6.474	3.47	-1.059	1.756	2.13	0.186	2.744	3.896
	Q8L3R3:	-0.532	4.004	-0.901	-1.057	1.65	2.161	0.206	2.516	3.995
	Q42484:	-2.272	4.465	1.861	-0.93	1.753	2.131	0.31	2.64	3.962
	Q9FG90:	0.561	1.199	-2.914	-0.998	1.737	2.192	0.304	2.805	3.878
	P60839:	-0.875	0.27	-3.259	-1.019	1.492	2.226	0.341	2.36	3.979
	Q9LMP6:	-2.012	2.658	-2.44	-1.011	1.749	2.173	0.311	2.622	4.027
	Q9SI85:	1.988	2.189	0.533	-1.097	1.569	2.303	0.227	2.686	3.97
	A0A654FPA2:	-1.696	0.917	-2.399	-0.979	1.521	2.249	0.338	2.288	4.111
	Q8RXS5:	0.769	-1.416	-1.301	-1.027	1.984	2.028	0.289	3.047	3.755
	Q9FLB4:	-1.267	4.808	-0.481	-1.106	1.746	2.057	0.069	2.974	3.73
	O82484:	-1.933	3.407	-1.447	-1.213	1.782	2.178	0.068	2.882	3.868
	P60838:	0.296	3.727	-0.523	-0.933	1.558	2.213	0.509	2.527	3.876
	A0A1P8AP86:	0.346	3.726	-1.011	-1.01	1.654	2.176	0.294	2.549	3.978
	A0A5S9WIX4:	-1.385	2.512	-2.046	-1.05	1.51	2.244	0.223	2.375	4.081
	Q9LVT3:	-1.845	3.448	-1.154	-0.728	1.882	2.18	0.626	3.001	3.728
Non A. Thaliana	Q6WSR5:	-1.979	4.043	2.237	-1.007	1.931	1.948	0.219	3.24	3.359
	Q6WWJ4:	-2.775	4.1	2.737	-1.383	2.093	2.077	-0.525	3.414	3.774
	Q9S7Q4:	-2.394	3.349	2.366	-1.141	1.448	2.293	-0.013	1.944	4.867
	Q15J16:	-1.954	3.759	2.542	-0.989	1.718	2.198	-0.02	2.844	3.941
	**Alphafold2 - Inactive Control**									
EDVID	Q38834:	0.07	4.331	-0.514	-0.966	2.495	-0.892	-2.833	3.478	-1.77
	Q8W4J9:	-0.697	4.714	-0.534	-0.955	2.481	-0.552	-3.075	3.601	-1.03
	Q8W3K3:	-0.697	4.791	-0.863	-1.005	2.547	-0.777	-3.125	3.489	-1.897
	Q9LQ54:	-0.894	4.708	-0.677	-1.031	2.524	-0.875	-3.21	3.386	-1.645
	A0A654EJC3:	-0.574	4.763	-0.778	-0.961	2.53	-0.867	-3.075	3.419	-2.032
	Q9SX38:	-0.809	4.819	-0.049	-0.989	2.637	-0.419	-3.074	3.907	-1.824
	Q8W474:	-0.656	4.843	-0.805	-0.999	2.575	-0.876	-3.096	3.64	-1.492
	Q9C646:	-0.585	4.799	-0.829	-0.989	2.527	-0.802	-3.08	3.57	-1.423
	Q8W3K0:	-0.966	4.776	-0.966	-1.138	2.49	-0.861	-3.332	3.407	-1.432
	Q9XIF0:	-0.567	4.53	-0.646	-1.031	2.523	-0.755	-3.215	3.496	-1.271
	Q9STE7:	-0.735	4.922	-0.78	-0.861	2.545	-0.683	-3.029	3.59	-1.66
	A0A654EJG6:	-0.878	4.777	-1.195	-1.048	2.505	-0.919	-3.195	3.302	-1.741
	Q9M667:	-0.828	4.841	-0.941	-0.938	2.516	-0.715	-3.032	3.491	-1.601
	Q39214:	-0.638	4.751	-0.633	-0.951	2.518	-0.742	-3.019	3.266	-1.964
	Q9LRR4:	-0.838	4.728	-1.045	-0.887	2.593	-0.766	-3.022	3.444	-1.516
	Q9STE5:	-0.65	4.805	-1.196	-0.805	2.491	-0.861	-2.987	3.457	-1.894
RPS5	O64973:	-0.327	4.545	-0.858	-1.087	2.513	-0.898	-3.286	3.377	-1.517
	Q940K0:	0.093	1.804	-4.527	-1.057	2.628	-0.732	-3.22	3.601	-1.047
	Q8L3R3:	-1.445	1.05	-4.732	-1.026	2.598	-0.643	-3.224	3.573	-0.954
	Q42484:	2.303	1.977	-2.603	-0.919	2.669	-0.641	-3.11	3.585	-1.19
	Q9FG90:	1.013	0.111	-2.977	-1.17	2.651	-0.785	-3.361	3.531	-1.608
	P60839:	-4.609	0.511	-2.686	-1.042	2.591	-0.725	-3.253	3.559	-1.421
	Q9LMP6:	-5.259	0.405	-1.827	-0.838	2.42	-1.037	-2.97	3.397	-1.686
	Q9SI85:	-3.538	1.625	1.044	-1.105	2.452	-0.976	-3.231	3.373	-1.565
	A0A654FPA2:	-0.92	-1.564	-2.624	-1.063	2.597	-0.776	-3.201	3.602	-1.195
	Q8RXS5:	-0.576	3.12	-2.52	-1.007	2.462	-1.046	-3.129	3.352	-1.872
	Q9FLB4:	0.773	0.804	-3.174	-1.056	2.538	-0.771	-3.044	3.865	-2.157
	O82484:	0.496	0.244	-3.222	-1.048	2.547	-0.887	-3.174	3.516	-1.435
	P60838:	0.644	2.914	-2.614	-1.007	2.573	-0.785	-3.181	3.568	-1.141
	A0A1P8AP86:	-0.669	5.291	0.434	-1.082	2.49	-0.871	-3.215	3.445	-1.459
	A0A5S9WIX4:	0.149	0.728	-3.386	-1.193	2.729	-0.582	-3.472	3.655	-0.897
	Q9LVT3:	-0.982	1.095	3.548	-1.31	2.815	-0.671	-3.592	3.258	-0.891
Non A. Thaliana	Q6WSR5:	-0.595	4.698	-1.587	-0.833	2.405	-1.016	-2.827	3.503	-1.373
	Q6WWJ4:	-1.623	4.965	-0.895	-1.343	2.826	-0.925	-3.554	3.198	-1.558
	Q9S7Q4:	-5.605	1.871	0.602	-0.919	2.565	-0.742	-3.187	3.735	-1.857
	Q15J16:	0.212	4.527	-0.404	-1.018	2.44	-1.016	-2.967	3.458	-1.904

	RoseTTAFold - ADP									
EDVID	Q38834:	-0.51	4.113	-2.083	-0.869	2.256	-1.188	-2.645	2.931	-2.484
	Q8W4J9:	-0.745	4.343	-1.291	-0.975	2.308	-0.894	-3.052	3.149	-1.905
	Q8W3K3:	-0.552	4.135	-1.69	-0.804	2.234	-1.094	-2.768	2.831	-2.761
	Q9LQ54:	-2.539	3.208	-1.324	-1.387	1.352	-0.595	-3.808	0.98	-1.237
	A0A654EJC3:	-0.555	4.2	-1.701	-0.75	2.214	-1.172	-2.851	2.764	-2.673
	Q9SX38:	-0.591	3.92	-2.128	-0.888	2.155	-1.112	-2.548	2.482	-3.05
	Q8W474:	-0.758	4.251	-1.619	-0.871	2.265	-1.183	-2.971	2.844	-2.233
	Q9C646:	-0.618	4.321	-1.423	-0.937	2.306	-1.036	-3.047	3.14	-1.986
	Q8W3K0:	-1.781	2.844	-2.378	-1.306	1.261	-0.938	-3.408	0.72	-2.045
	Q9XIF0:	-0.461	4.162	-1.461	-0.938	2.237	-1.033	-3.014	3.15	-1.979
	Q9STE7:	-0.731	4.196	-1.847	-0.808	2.215	-1.135	-2.744	2.595	-2.721
	A0A654EJG6:	-0.617	4.319	-1.713	-0.867	2.229	-1.161	-2.975	2.919	-2.166
	Q9M667:	-0.821	4.246	-1.786	-0.85	2.272	-1.209	-2.714	2.658	-2.655
	Q39214:	-0.681	3.792	-2.203	-0.861	1.956	-1.274	-2.922	1.812	-2.843
	Q9LRR4:	-0.447	3.885	-2.432	-0.661	2.091	-1.374	-2.568	2.397	-2.777
	Q9STE5:	-0.552	4.042	-2.023	-0.644	2.124	-1.278	-2.622	2.427	-2.887
RPS5	O64973:	0.213	0.005	-1.188	-1.04	2.124	-0.914	-3.361	2.175	-1.648
	Q940K0:	-0.228	0.083	-4.56	-1.131	2.285	-0.343	-3.501	2.743	-0.627
	Q8L3R3:	-0.929	-2.341	-0.121	-0.772	2.307	-1.139	-2.877	3.089	-2.085
	Q42484:	-0.94	1.421	-1.421	-1.265	2.076	-0.895	-3.065	0.892	-1.997
	Q9FG90:	-3.586	-2.108	0.864	-0.817	2.478	-0.939	-2.96	3.213	-1.743
	P60839:	-1.136	-2.784	0.607	-0.787	2.354	-1.14	-2.884	3.123	-2.016
	Q9LMP6:	-1.143	2.788	-2.138	-0.722	2.349	-1.153	-2.784	3.067	-2.2
	Q9SI85:	-1.095	-2.186	0.063	-0.823	2.2	-1.261	-2.884	2.762	-2.172
	A0A654FPA2:	-3.074	-3.578	0.412	-0.823	2.343	-1.213	-2.909	3.006	-2.21
	Q8RXS5:	-0.902	-2.765	-0.417	-0.898	2.386	-1.088	-3.065	2.872	-2.103
	Q9FLB4:	1.58	1.954	-2.539	-0.963	2.482	-0.873	-3.134	3.236	-1.695
	O82484:	1.589	-1.224	-1.526	-0.952	2.314	-0.957	-3.248	2.872	-1.575
	P60838:	-1.336	-3.777	-1.133	-0.693	2.162	-1.254	-2.719	2.892	-2.344
	A0A1P8AP86:	-0.96	-2.237	0.976	-0.77	2.217	-1.295	-2.792	2.952	-2.308
	A0A5S9WIX4:	1.654	-0.323	-0.358	-1.011	2.354	-0.93	-3.24	2.867	-1.756
	Q9LVT3:	2.083	1.841	-1.959	-0.858	2.619	-0.858	-3.047	3.352	-1.413
Non A. Thaliana	Q6WSR5:	-0.185	3.365	-2.866	-0.635	1.911	-1.406	-2.438	2.501	-2.681
	Q6WWJ4:	-2.201	4.064	-2.295	-1.474	2.56	-1.31	-3.323	2.064	-2.669
	Q9S7Q4:	-0.493	3.968	-1.871	-0.772	2.162	-1.263	-2.949	2.407	-3.011
	Q15J16:	-0.992	4.267	-1.289	-1.03	2.312	-1.071	-2.88	3.069	-2.217

	RoseTTAFold - ATP									
EDVID	Q38834:	-0.481	3.847	-2.472	-0.902	2.073	-1.341	-2.592	2.512	-2.839
	Q8W4J9:	-0.589	4.341	-1.364	-0.937	2.349	-0.905	-3.059	3.27	-1.744
	Q8W3K3:	-0.479	4.246	-1.762	-0.825	2.234	-1.148	-2.713	2.873	-2.792
	Q9LQ54:	-1.411	4.287	-1.716	-0.89	2.269	-1.173	-3.121	2.689	-2.292
	A0A654EJC3:	-0.531	4.203	-1.453	-0.821	2.236	-1.087	-2.976	2.959	-2.417
	Q9SX38:	-0.584	4.387	-1.673	-0.875	2.345	-1.004	-2.885	3.104	-2.768
	Q8W474:	-0.584	4.326	-1.684	-0.814	2.304	-1.214	-2.934	2.965	-2.184
	Q9C646:	-0.604	4.273	-1.536	-0.9	2.288	-1.052	-3.016	3.101	-1.991
	Q8W3K0:	-1.11	3.787	-2.073	-0.919	1.773	-1.284	-2.877	1.879	-2.795
	Q9XIF0:	-0.384	4.182	-1.398	-0.87	2.256	-1.065	-2.989	3.096	-1.98
	Q9STE7:	-0.91	4.295	-1.703	-0.836	2.269	-1.125	-2.807	2.623	-2.697
	A0A654EJG6:	-0.709	4.426	-1.495	-0.886	2.29	-1.114	-3.049	2.953	-2.018
	Q9M667:	-0.739	4.282	-1.713	-0.872	2.298	-1.137	-2.782	2.825	-2.479
	Q39214:	-0.438	3.927	-2.027	-0.761	2.023	-1.222	-2.789	2.118	-2.791
	Q9LRR4:	-0.58	3.932	-2.283	-0.704	2.122	-1.302	-2.625	2.387	-2.683
	Q9STE5:	-0.47	4.094	-1.926	-0.609	2.149	-1.239	-2.686	2.624	-2.674
RPS5	O64973:	-2.297	-3.999	-1.159	-0.72	2.313	-1.208	-2.75	3.113	-2.221
	Q940K0:	-2.413	-3.043	-3.978	-0.731	2.448	-1.068	-2.834	3.254	-1.958
	Q8L3R3:	-2.509	-3.166	0.603	-0.754	2.385	-1.092	-2.852	3.175	-1.987
	Q42484:	-1.371	2.041	-2.761	-1.318	2.148	-0.72	-3.086	0.571	-1.312
	Q9FG90:	-1.705	1.002	0.305	-0.811	2.483	-1.04	-2.999	3.078	-1.845
	P60839:	-1.841	-0.606	1.844	-0.869	2.301	-1.104	-3.007	2.858	-2.071
	Q9LMP6:	-0.398	-2.443	-3.178	-0.771	2.415	-1.099	-2.931	3.167	-1.882
	Q9SI85:	-3.049	-0.293	-4.162	-0.831	2.299	-1.292	-2.893	2.786	-2.273
	A0A654FPA2:	-2.83	-3.384	-2.809	-0.771	2.353	-1.21	-2.83	3.053	-2.22
	Q8RXS5:	1.207	-0.638	0.259	-1.414	2.438	-0.098	-3.75	2.458	0.11
	Q9FLB4:	2.289	2.829	-2.838	-0.886	2.489	-0.917	-3.071	3.307	-1.714
	O82484:	0.733	-2.872	-1.207	-0.884	2.516	-0.966	-3.036	3.441	-1.585
	P60838:	-1.673	-3.478	-0.78	-0.75	2.415	-1.124	-2.841	3.347	-1.945
	A0A1P8AP86:	-0.141	1.127	1.916	-1.113	2.172	-0.78	-3.415	2.144	-1.431
	A0A5S9WIX4:	1.704	2.299	-2.885	-1.022	2.244	-0.89	-3.239	2.773	-1.639
	Q9LVT3:	2.049	1.616	-2.322	-0.803	2.637	-0.801	-3.011	3.384	-1.219
Non A. Thaliana	Q6WSR5:	-0.002	3.49	-2.658	-0.5	1.873	-1.429	-2.352	2.725	-2.5
	Q6WWJ4:	-1.512	4.338	-2.177	-1.26	2.558	-1.215	-3.362	2.636	-2.173
	Q9S7Q4:	-0.667	4.038	-1.633	-0.822	2.178	-1.193	-3.126	2.481	-2.825
	Q15J16:	-0.853	4.254	-1.426	-0.938	2.278	-1.151	-2.753	3.03	-2.363

	RoseTTAFold - No Ligand									
EDVID	Q38834:	-0.564	4.486	-1.301	-0.869	2.319	-1.044	-2.635	3.179	-2.278
	Q8W4J9:	-0.835	4.549	-0.835	-1.049	2.386	-0.873	-3.174	3.376	-1.619
	Q8W3K3:	-0.701	4.457	-1.186	-1.035	2.328	-1.043	-3.092	3.274	-2.361
	Q9LQ54:	-1.848	4.188	-0.505	-1.366	1.961	-0.831	-3.728	2.609	-1.353
	A0A654EJC3:	-0.817	4.429	-1.253	-0.948	2.303	-1.155	-3.083	3.042	-2.517
	Q9SX38:	-0.62	4.432	-1.117	-0.953	2.317	-0.938	-2.734	3.08	-2.808
	Q8W474:	-0.932	4.503	-1.172	-1.081	2.382	-1.073	-3.257	3.181	-1.825
	Q9C646:	-0.815	4.451	-0.998	-1.009	2.359	-1.032	-3.138	3.254	-1.84
	Q8W3K0:	-1.302	4.46	-1.188	-1.231	2.281	-1.033	-3.485	2.918	-1.837
	Q9XIF0:	-0.579	4.298	-0.983	-1.012	2.331	-1.024	-3.12	3.319	-1.794
	Q9STE7:	-0.993	4.446	-1.257	-0.971	2.357	-1.077	-3.015	2.937	-2.544
	A0A654EJG6:	-0.868	4.518	-1.072	-1.023	2.355	-1.039	-3.185	3.15	-1.903
	Q9M667:	-0.854	4.372	-1.016	-0.99	2.317	-1.051	-2.92	3.069	-2.347
	Q39214:	-0.973	4.329	-1.111	-1.01	2.306	-1.053	-3.099	2.688	-2.614
	Q9LRR4:	-1.823	4.573	0.104	-1.756	2.501	0.142	-3.99	3.267	0.653
	Q9STE5:	-0.972	4.297	-1.439	-0.874	2.224	-1.223	-2.898	2.759	-2.819
RPS5	O64973:	-0.905	-2.184	-3.611	-0.918	2.567	-0.883	-2.981	3.641	-1.567
	Q940K0:	-0.531	-0.735	-4.202	-0.931	2.619	-0.908	-3.048	3.497	-1.75
	Q8L3R3:	-2.025	-1.988	-3.564	-0.852	2.521	-1.031	-2.929	3.383	-1.999
	Q42484:	-0.554	2.192	-1.594	-1.973	2.368	-0.503	-3.696	0.669	-1.045
	Q9FG90:	-1.986	-2.775	-1.935	-0.871	2.615	-0.874	-3.032	3.469	-1.669
	P60839:	-1.515	-0.773	-3.944	-0.964	2.626	-0.908	-3.12	3.508	-1.621
	Q9LMP6:	-1.584	-2.753	-3.553	-0.817	2.568	-0.986	-2.919	3.473	-1.878
	Q9SI85:	-4.287	0.426	-3.907	-0.877	2.412	-1.115	-2.948	3.222	-1.911
	A0A654FPA2:	-1.747	-1.368	-4.041	-0.85	2.487	-1.106	-2.923	3.395	-2.024
	Q8RXS5:	-0.996	-2.378	-2.873	-0.876	2.554	-0.99	-2.927	3.285	-2.072
	Q9FLB4:	-0.088	-0.78	-3.4	-0.918	2.588	-0.958	-3.072	3.202	-2.026
	O82484:	-0.112	-0.312	-2.791	-0.966	2.583	-0.9	-3.048	3.603	-1.605
	P60838:	-0.191	-1.893	-2.483	-0.788	2.493	-0.908	-2.843	3.583	-1.648
	A0A1P8AP86:	-0.663	-1.535	-1.831	-0.972	2.455	-1.151	-3.094	3.26	-2.06
	A0A5S9WIX4:	-1.144	-1.465	-3.496	-0.969	2.649	-0.752	-3.02	3.803	-1.325
	Q9LVT3:	0.317	0.023	-1.311	-0.772	2.667	-1.08	-2.968	3.182	-1.944
Non A. Thaliana	Q6WSR5:	-0.789	4.37	-1.367	-0.815	2.205	-1.07	-2.869	3.188	-1.658
	Q6WWJ4:	-2.065	4.536	-1.645	-1.349	2.61	-1.261	-3.51	2.682	-2.233
	Q9S7Q4:	-0.702	4.277	-1.173	-0.96	2.298	-1.111	-3.325	2.93	-2.622
	Q15J16:	-1.007	4.513	-0.601	-1.082	2.493	-0.917	-3.03	3.613	-1.621

	Omegafold									
EDVID	Q38834:	-0.093	4.792	-1.18	-0.607	2.659	-1.009	-2.437	3.531	-2.053
	Q8W4J9:	-0.117	4.919	-1.347	-0.568	2.65	-0.865	-2.591	3.853	-1.471
	Q8W3K3:	-0.091	5.004	-0.92	-0.556	2.72	-0.911	-2.556	3.955	-2.037
	Q9LQ54:	-0.633	5.102	-0.642	-0.761	2.729	-0.774	-2.966	3.727	-1.292
	A0A654EJC3:	0.075	4.998	-0.604	-0.504	2.782	-0.716	-2.564	4.125	-1.574
	Q9SX38:	-0.628	5.022	-0.778	-0.8	2.731	-0.667	-3.026	3.944	-2.15
	Q8W474:	-0.064	5.149	-0.591	-0.57	2.813	-0.77	-2.563	4.198	-1.079
	Q9C646:	-0.233	5.079	-0.221	-0.663	2.819	-0.586	-2.713	4.169	-0.797
	Q8W3K0:	-0.157	4.784	-1.1	-0.65	2.64	-0.995	-2.788	3.886	-1.555
	Q9XIF0:	0.066	4.623	-0.928	-0.515	2.626	-0.91	-2.648	3.729	-1.363
	Q9STE7:	-0.679	5.158	-0.978	-0.69	2.778	-0.633	-2.921	3.71	-1.593
	A0A654EJG6:	-0.082	4.962	-1.506	-0.702	2.709	-1.062	-2.684	3.903	-1.877
	Q9M667:	-0.488	5.072	-0.939	-0.698	2.715	-0.736	-2.797	3.649	-1.594
	Q39214:	-2.159	4.198	2.254	-1.295	2.286	2.142	-0.46	2.36	4.539
	Q9LRR4:	-0.247	5.03	-0.243	-0.516	3.001	-0.586	-2.558	4.164	-0.986
	Q9STE5:	-0.772	4.977	-1.507	-0.716	2.663	-0.986	-2.945	3.357	-2.154
RPS5	O64973:	-5.439	-0.596	-3.328	-0.785	2.941	-0.425	-2.885	4.153	-0.079
	Q940K0:	-4.653	-2.476	-2.045	-0.721	2.963	-0.459	-2.834	4.159	-0.298
	Q8L3R3:	-1.483	-2.83	-4.074	-0.764	2.909	-0.498	-2.942	4.038	-0.37
	Q42484:	-4.818	1.374	-3.212	-0.599	2.978	-0.771	-2.803	4.012	-1.225
	Q9FG90:	-4.714	-2.425	-0.882	-0.7	2.799	-0.753	-2.863	3.911	-0.866
	P60839:	-0.863	3.408	-4.952	-0.918	2.896	-0.335	-3.027	3.911	0.226
	Q9LMP6:	-5.465	0.306	-2.112	-0.537	2.888	-0.886	-2.718	3.998	-1.057
	Q9SI85:	-5.008	-0.517	-2.648	-0.745	2.849	-0.809	-2.783	3.978	-0.982
	A0A654FPA2:	-1.492	-2.314	-4.603	-0.747	2.943	-0.364	-2.858	4.083	-0.035
	Q8RXS5:	-4.945	0.348	-3.108	-0.719	2.837	-0.79	-2.834	4.125	-1.114
	Q9FLB4:	-6.02	-0.218	-3.264	-1.088	2.909	-0.299	-3.363	3.868	-0.22
	O82484:	-0.626	5.222	-2.958	-0.896	2.892	-0.388	-3.076	3.949	-0.103
	P60838:	-4.068	-0.732	-4.551	-0.779	2.901	-0.307	-2.977	3.938	0.125
	A0A1P8AP86:	-0.659	4.709	-3.922	-0.897	2.863	-0.327	-2.923	4.189	0.09
	A0A5S9WIX4:	-4.596	-2.308	-0.475	-0.948	2.958	-0.299	-3.121	3.991	0.008
	Q9LVT3:	0.457	5.656	-1.785	-1.462	2.991	-1.159	-3.819	3.278	-1.184
Non A. Thaliana	Q6WSR5:	-1.925	3.95	2.385	-0.779	2.017	1.975	0.44	3.429	3.346
	Q6WWJ4:	-3.337	4.284	1.213	-1.726	2.772	1.511	-1.973	3.786	3.583
	Q9S7Q4:	-0.576	4.71	-1.028	-0.939	2.75	-0.866	-3.44	3.744	-1.893
	Q15J16:	-0.958	4.753	-0.857	-0.951	2.685	-0.872	-2.974	3.585	-1.571
